# The “Yin and Yang” of Natural Compounds in Anticancer Therapy of Triple-Negative Breast Cancers

**DOI:** 10.3390/cancers10100346

**Published:** 2018-09-21

**Authors:** Elizabeth Varghese, Samson Mathews Samuel, Mariam Abotaleb, Sohaila Cheema, Ravinder Mamtani, Dietrich Büsselberg

**Affiliations:** 1 Department of Physiology and Biophysics, Weill Cornell Medicine-Qatar, Education City, Qatar Foundation, Doha 24144, Qatar; elv2007@qatar-med.cornell.edu (E.V.); sms2016@qatar-med.cornell.edu (S.M.S.); mariam.abotaleb@aucegypt.edu (M.A.); 2 Institute for Population Health, Weill Cornell Medicine-Qatar, Education City, Qatar Foundation, Doha 24144, Qatar; soc2005@qatar-med.cornell.edu (S.C.); ram2026@qatar-med.cornell.edu (R.M.)

**Keywords:** anticancer therapy, apoptosis, chemotherapy, natural compounds, signaling pathways, triple-negative breast cancers

## Abstract

Among the different types of breast cancers, triple-negative breast cancers (TNBCs) are highly aggressive, do not respond to conventional hormonal/human epidermal growth factor receptor 2 (HER2)-targeted interventions due to the lack of the respective receptor targets, have chances of early recurrence, metastasize, tend to be more invasive in nature, and develop drug resistance. The global burden of TNBCs is increasing regardless of the number of cytotoxic drugs being introduced into the market each year as they have only moderate efficacy and/or unforeseen side effects. Therefore, the demand for more efficient therapeutic interventions, with reduced side effects, for the treatment of TNBCs is rising. While some plant metabolites/derivatives actually induce the risk of cancers, many plant-derived active principles have gained attention as efficient anticancer agents against TNBCs, with fewer adverse side effects. Here we discuss the possible oncogenic molecular pathways in TNBCs and how the purified plant-derived natural compounds specifically target and modulate the genes and/or proteins involved in these aberrant pathways to exhibit their anticancer potential. We have linked the anticancer potential of plant-derived natural compounds (luteolin, chalcones, piperine, deguelin, quercetin, rutin, fisetin, curcumin, resveratrol, and others) to their ability to target multiple dysregulated signaling pathways (such as the Wnt/β-catenin, Notch, NF-κB, PI3K/Akt/mammalian target of rapamycin (mTOR), mitogen-activated protein kinase (MAPK) and Hedgehog) leading to suppression of cell growth, proliferation, migration, inflammation, angiogenesis, epithelial-mesenchymal transition (EMT) and metastasis, and activation of apoptosis in TNBCs. Plant-derived compounds in combination with classical chemotherapeutic agents were more efficient in the treatment of TNBCs, possibly with lesser side effects.

## 1. Background and Current Understanding

Cancer is the second leading cause of death globally with 8.8 million cancer-related deaths in 2015. Cancer of lung (1.69 million deaths), liver (788,000 deaths), colon/rectum (774,000 deaths), stomach (754,000 deaths), and breast (571,000 deaths) are the most common cancers [1]. Worldwide, the incidence of cancer will increase within the next two decades by 50%, from 14 million to 21 million and cancer-related deaths will rise by 60%, from 8 million to 13 million. Africa, Asia, and South America will be most affected. Nevertheless, statistical reports of the most common cancers from North America showed a decrease in cancer-related death incidence by 11–13% between 2010 and 2014 due to improvements in anticancer therapy [2].

Breast cancer is the most common cancer among women worldwide and highest in women between 44 and 55 years of age. Although equally prevalent in developed and developing countries, breast cancer survival rates are higher (80%) in developed countries when compared to developing countries (less than 60%) and underdeveloped countries (less than 40%) [3]. Besides hormonal status, hormonal therapy, sedentary life styles, excessive alcohol consumption, and certain dietary habits, some pre-disposing genetic factors such as BRCA1, BRCA2, and p53 mutations place women at a higher risk for the development of breast cancer [4]. Interestingly, apart from the genetic factors, most other risk factors are based on lifestyle.

Over the past few decades, advances in the knowledge of breast cancer, supported by cutting-edge research and advanced technologies in diagnosis and treatment, have significantly increased the survival rates among breast cancer subjects. The therapeutic interventions available for breast cancer are better focused and highly specific. Gene expression profiling has enabled the classification of breast cancer into different types and subtypes (explained in detail in the Section 2). Among all different types of breast cancers, triple-negative breast cancers (TNBCs) are highly aggressive and associated with poor prognosis [5,6,7,8,9,10]. Their lack of hormonal/HER2 receptors and a wide variation in molecular characteristics make TNBCs difficult to treat. Hence surgery, radiation, and different chemotherapeutic-cytotoxic drug combinations are currently used to treat TNBCs, but with little efficacy and higher chances of relapse, metastasis, and invasion [6,7,8,9,10,11,12]. There is, therefore, a lingering need to identify key biomarkers and molecular targets for therapeutic intervention and for the discovery of associated specific and efficient drugs/agents for the treatment of TNBCs. Using the Internet, a layperson can access information on herbal/plant-based remedies for different diseases, including cancer, at the click of a button. It is the duty and responsibility of the scientific research community to filter this online information through focused and thorough experimental approaches to provide the best possible scientifically validated data, with the primary aim of reminding the public that “prevention is better than cure”, while also aiming at improving the quality of life of affected individuals.

Several epidemiological studies reported an association between the reduced risk of cancer and the consumption of certain protective phytochemicals, minerals, and anti-oxidants [13,14,15]. We have reported the anti-neoplastic and tumor suppressive effects of clove buds and oregano in breast cancer cells [16,17]. Additionally, several extensive in vitro, in vivo, preclinical, and a handful of clinical studies have reported on the anticancer potential of certain plant-derived active principles such as curcumin, resveratrol, and others [18,19,20,21]. Paclitaxel, camptothecin, vinblastine, vincristine, topotecan, and other such drugs developed from plant sources are widely marketed and were successfully used as effective anticancer drugs [22]. Naturally occurring phytochemicals can target and modulate the oncogenic, anti-apoptotic, epithelial-mesenchymal transition (EMT) and metastasis related signaling mechanisms, such as the Wnt [23], Notch [24], NF-κB [25], PI3K/Akt/mTOR [26], MAPK [27], and Hedgehog [28] pathways in TNBCs [29]. Natural compounds such as luteolin, deguelin, chalcones, piperine, maximiscin, curcumin, and resveratrol gained attention and importance as anticancer agents owing to their safety, lesser adverse side effects, and efficacy to target multiple signaling pathways in cancers, including TNBCs. Herein we focus on TNBCs and their relevant signaling pathways and review and discuss the potential beneficial effects of plant-derived compounds in the treatment of TNBCs, while acknowledging that certain food derivatives/dietary components (such as asparagine) could increase the risk of cancers and aggravate cancer complications.

## 2. Types and Subtypes of Breast Cancer

Breast cancer is a heterogeneous disease with wide variation in gene expression [30]. This heterogeneity determines the course of the disease, the outcome of treatment, and patient survival. Breast cancers are broadly classified into two groups based on the absence or presence of receptors in their molecular signature, the status of which determines the type of therapeutic intervention [31]. The presence of one or a combination of the estrogen receptor (ER), progesterone receptor (PR), or human epidermal growth factor receptor 2 (HER2) render one group of breast cancers susceptible to hormonal/HER2 targeted therapy [32]. On the other hand, breast cancers devoid of any of these three receptors are classified as TNBCs [33,34]. Thus, receptor status has a major implication on therapy and prognosis among breast cancer patients.

TNBCs account for 10–24% of all breast cancers [35]. Based on gene ontologies and gene expression analysis, TNBCs are further classified into six different molecular subtypes: (1) basal-like 1 (BL1), (2) basal-like 2 (BL2), (3) immunomodulatory (IM), (4) mesenchymal-like (M), (5) mesenchymal stem-like (MSL), and (6) luminal androgen receptors (LAR) subtypes. Table 1 shows the molecular subtyping of TNBC based on distinct gene ontology and differential gene expression. “Basal-like” is the most common type, representing 70–80% of all subtypes of TNBC [6].

Due to the absence of hormonal/HER2 receptors, TNBCs do not respond to conventional hormonal/HER2 targeted therapies, making them highly aggressive [36]. Hence, chemotherapy using cytotoxic drugs is generally used in the treatment of TNBCs [8,9,12]. TNBCs often exhibit metastasis to secondary sites such as the lung and brain [37]. Moreover, they are highly proliferative and regularly develop resistance against chemotherapeutic agents [36]. The chemotherapeutic agents commonly used for treatment of TNBCs include taxanes and anthracyclines. Platinum-based compounds (cisplatin and carboplatin) have long been used for the treatment of breast cancers and were the first line of treatment for metastatic breast cancer [12,38]. Frequently, they are also used for treating early stage TNBC as it was proven to be effective in TNBCs with lung metastasis [31]. Identifying a specific anticancer agent for the treatment of TNBCs is challenging because of their heterogeneous molecular portrait.

## 3. Aberrant Pathways in Breast Cancer/TNBCs

**Figure 1:**
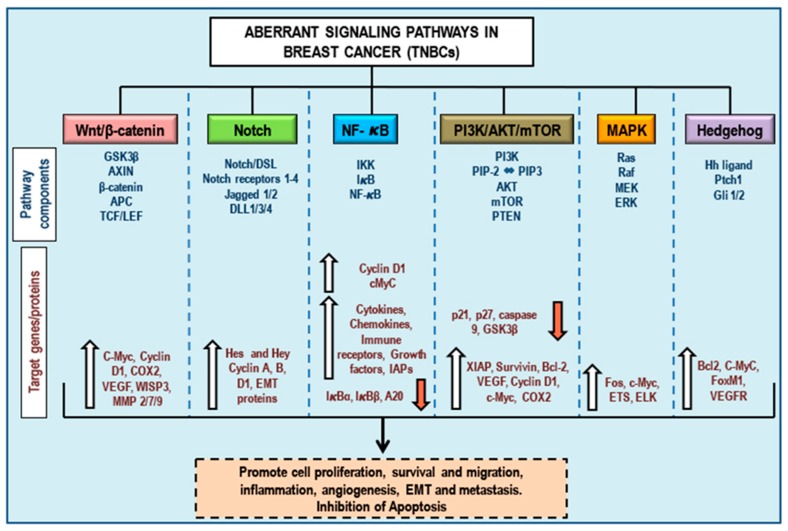
Oncogenic signaling in triple-negative breast cancers (TNBCs). Aberrant signaling pathways (Wnt/β-catenin, Notch, NF-κB, PI3K/AkT/mTOR, MAPK and Hedgehog) significant in TNBCs and specific pathway-associated components, target genes, and the dysregulated proteins involved in the various signaling pathways leading to cancer progression by supporting cell proliferation, survival and migration, inflammation, angiogenesis, epithelial-mesenchymal transition (EMT) and metastasis, and inhibition of apoptosis. The upward white arrows indicate activation/upregulation while the downward red arrows indicate inhibition/downregulation.

A hallmark of cancer is its heterogeneity at the gene and protein level. Cancer is characterized by a deregulation of several cell signaling pathways including those that regulate apoptosis, proliferation, cell cycle, immune response, angiogenesis, and migration. Hence, drugs designed to cure cancer basically target one or more of these signaling pathways. In TNBCs, six major cell signaling pathways are de-regulated: (1) Wnt, (2) Notch, (3) NF-κB, (4) PI3K/Akt/mTOR, (5) MAPK, and (6) Hedgehog pathways. Figure 1 shows the oncogenic signaling, pathway-associated components, target genes, and dysregulated proteins involved in cancer growth and progression in TNBCs. Several plant-derived active compounds were identified (see next Section 4) which target and modulate these oncogenic pathways, thereby indicating the potential of using bioactive phytochemicals in the treatment of TNBCs.

### 3.1. Wnt/β-Catenin Signaling and TNBCs

Wnt/β-catenin modulates embryonic growth and regulates cell migration, proliferation, differentiation, and survival [23]. In the absence of Wnt ligands, the cytoplasmic form of β-catenin (which is also a cell membrane-bound protein) is sequestered in a death complex comprising of the tumor suppressor, adenomatous polyposis coli (APC), axin, glycogen synthase kinase-3β (GSK-3β), and casein kinase 1 (CK1) [39]. The kinases in this death complex (GSK-3β and CK1) in turn phosphorylate β-catenin, tagging it for subsequent poly-ubiquitination and degradation through the 26S proteasome pathway [39]. On the contrary, the presence of Wnt proteins (secreted glycoproteins) and their subsequent formation of a ternary complex with its membrane receptor, the frizzled (mainly FZD_7_) receptor (a seven-pass transmembrane receptor) and a co-receptor, the low-density lipoprotein receptor-related protein (LRP5/6), activates the signaling pathway [39,40]. This Wnt-receptor association leads to GSK-3β inactivation, as well as release of β-catenin from the death complex and its translocation into the nucleus, driving the expression of T-cell factor/lymphoid enhancing factor (TCF/LEF) target genes. This subsequently leads to gene translation and expression of proteins (such as c-Myc, cyclin D1, COX2, VEGF, MMP7 and WISP3) [41] (Figure 1) that regulate cell cycle, migration, proliferation, differentiation, and survival [39,42]. GSK-3β also mediates other major signaling pathways such as the Notch pathway, the NF-κB signaling pathway, the PI3K pathway, and the Hedgehog pathway [43].

While the Wnt/β-catenin signaling is implicated in several stages of the breast development [44,45], several scientific reports provide evidence indicating the abnormal activation of the Wnt/β-catenin signaling in breast cancers including TNBCs [46,47,48,49]. Microarray studies and meta-analysis data showed an activation of the Wnt signaling mechanism in TNBCs [41,50]. Both the FZD_7_ receptor and the LRP5/6 co-receptor, required for the activation of Wnt signaling, were upregulated in TNBCs, while knockdown of the FZD_7_ or LRP6 in TNBC cells suppressed Wnt/β-catenin signaling, cell proliferation, and tumor growth in vivo [23,40,51]. Wnt signaling promotes EMT and stemness in HER2 overexpressing breast cancers [52], which also may be true for TNBCs. Wnt signaling is also implicated in lung and bone metastasis of breast cancer [53]. Wnt/β-catenin signaling dependent activation of target cyclin D1 is associated with poor prognosis in breast cancer patients [54]. Additionally, the activation of Wnt/β-catenin signaling correlated with the invasiveness of TNBCs and is associated with a negative clinical outcome [55,56]. Blocking the Wnt/β-catenin pathway suppresses breast cancer metastasis by inhibition of stemness [57]. The Wnt signaling mechanism is also responsible for regulation of vasculogenic mimicry (endothelium-independent, non-angiogenic, blood perfused, microcirculatory system formed by tumor cells) in TNBCs [58]. This evidence suggests that the Wnt/β-catenin signaling mechanism plays a key role in the development of TNBC and progression of the disease, thereby providing a potential target for therapeutic intervention for the disease.

### 3.2. Notch Signaling and TNBCs

Notch signaling, one of the most evolutionarily conserved signaling pathways in physiology, regulates processes essential for embryonic and post-natal development and modulates a wide range of processes related to stem-cell maintenance, cell differentiation, proliferation, motility, and survival [59,60]. Canonical Notch signaling requires cell-to-cell contact and communication [60,61], the resultant signals being transduced to the nucleus to drive the expression of target genes [61]. In the mammalian system, one of the five Notch ligands (Jagged (JAG) 1 and 2 and Delta-like ligand (DLL) 1, 3, and 4) expressed on the surface of the signaling cells binds to the extracellular domain (ECD) on one of the four Notch receptors (Notch 1–4) on an adjacent signal receiving cell, thereby activating the receptor [24,59]. Ligand binding to a Notch receptor leads to metalloproteinase dependent cleavage of the ECD and subsequent degradation of the Notch extracellular truncation (NEXT) by γ-secretase [24,59,60,61,62]. The release of ECD from the activated Notch receptor triggers the release of the Notch intracellular domain (NICD) and its subsequent translocation of the NICD into the nucleus, followed by its interaction with co-activators Mastermind (MAM) and p300 to regulate the expression of Notch target genes [24,59,60,61,63,64]. The transcriptional targets include p21 and cyclin D1 (cell-cycle and apoptosis regulators), c-Myc and NF-κB (transcription factors), and VEGF and its receptors (angiogenesis) [65,66].

Normal breast development involves Notch signaling [65,67,68,69,70]. This supports the significant role for aberrant Notch signaling in the development of breast cancer. Notch receptors (Notch 1, 3 and 4) and ligands (JAG1 and DLL4) were highly expressed in human breast cancers, contributing to the abnormal growth of breast cancer cells, when compared to normal breast tissues obtained from the margin of the tumor section [59,71]. In TNBCs, the inhibition of Notch-4 significantly reduced Notch-4 dependent proliferation and invasiveness, leading to marked reduction in tumor volume and tumorigenicity [72,73]. Overexpression of Notch-1 receptor and ligand JAG1 supports the maintenance of breast cancer stem cells (CSCs) [74,75,76] and was crucial for cell survival, apoptotic inhibition, proliferation, adhesion, angiogenesis, metastatic potential, poor prognosis, relapse, and drug resistance in TNBCs [60,73,77]. Notch signaling in TNBCs (Figure 1) is critical for disease progression by triggering EMT [78]. The vacuolar-ATPase a2V subunit regulates Notch signaling and the antibody mediated neutralization of a2V hindered cell migration in TNBCs [79]. Notch-1 and 4, JAG1, and the downstream effector molecule, survivin, have prognostic significance in TNBCs [24]. In addition to the evidence suggesting that the Notch signaling pathway could be a potential target for therapeutic intervention in TNBCs, it is noteworthy that Notch signaling integrates signals from several other pathways, including Wnt signaling, Hedgehog signaling, VEGF/VEGFR2 signaling, PI3K/Akt/mTOR signaling, Ras/Raf signaling, NF-κB signaling, and HIF1-α dependent hypoxic signaling mechanisms [65,66,80].

### 3.3. NF-κB Signaling and TNBCs

The NF-κB signaling pathway is essential for several key biological processes, including differentiation during embryogenesis, inflammatory and immunological responses, cell proliferation, and survival [81,82,83]. The five identified members of the NF-κB family, the p65 (RelA), RelB, c-Rel, p50 (NF-κB1) and p52 (NF-κB2) proteins, share a highly conserved Rel homology domain (RHD) which is responsible for DNA binding, dimerization, and association with the repressor protein inhibitor of κB (IκB) [81,83,84]. NF-κB/Rel dimer is bound to the inhibitory protein IκB (IκBα being the most studied among the different IκB isoforms) in un-stimulated cells. The canonical NF-κB pathway is activated by extracellular stimuli (pro-inflammatory cytokines, growth factors) which are recognized by membrane-bound receptors (e.g., TNF receptor superfamily; TNFR, IL-1 receptor; IL-1R and Toll-like receptor 4; TLR4) that transmit the signal into the cell through adapter signaling proteins [82,83]. Subsequent activation of the IκB kinase (IKK) complex (comprised of IKKα, IKKβ, and regulatory NEMO/IKKγ proteins), specifically the active IKKβ, in turn mediates the phosphorylation and inactivation of the inhibitory IκBα and marks it for poly-ubiquitination and subsequent degradation through the 26S proteasome pathway, while the active NF-κB dimer complex is released from the inhibitory complex [82,83]. The active NF-κB dimer then translocates into the nucleus where it, either alone or in combination with other transcription factors including AP-1, ETS, and STAT, binds to target sequences and modulates the expression of target genes related to cell proliferation, survival, and apoptosis [82,83,85]. In addition to phosphorylation and inactivation of IκB proteins, IKK can mediate the crosstalk with other signaling pathways such as the MAPK and p53 pathways [85,86].

Activation of the NF-κB signaling pathway is implicated in different cancers, including breast cancers (Figure 1) [87]. The constitutive activation of NF-κB was reported in mainly in HER2 positive [88] and triple-negative [89,90] breast tumors and was responsible for cellular proliferation, angiogenesis, and evasion of apoptosis [82]. The NF-κB signaling mechanism is required for self-renewal and for the formation of xenograft tumors for TNBC cells [91]. In TNBCs, NF-κB inhibition and subsequent CD44 repression was correlated with the decrease in proliferation and invasiveness of TNBC cells [92]. Blocking NF-κB signaling using mutant IκB, MDA-MB-231 cells markedly reduced cell proliferation. NF-κB signaling controlled tumor proliferation, cell survival, and bone resorption in TNBCs and promoted bone tumor burden and tumor mediated osteolysis [93]. Additionally, coordinated autocrine expression of IL-6 and IL-8 stimulated the growth of TNBCs through NF-κB activation, conferred resistance to apoptosis and chemotherapy, and activated tumor growth [94]. Furthermore, NF-κB signaling is responsible for the protein “Morgana”, dependent TNBC cell invasion, and metastasis [95]. From the literature, it is evident that therapeutic approaches and anticancer phytochemical drugs that target NF-κB activation should prove to be efficient in the treatment of TNBCs.

### 3.4. PI3K/Akt/mTOR Signaling and TNBCs

The PI3K/Akt/mTOR pathway represents a key signaling mechanism responsible for cell survival proliferation, migration, metabolism, and drug resistance [96,97]. Knocking down either the α or β subunit of the catalytic p110 domain of PI3K (a heterodimer with p110 catalytic domain and regulatory p85 domain) leads to embryonic lethality in mice, indicating the critical role of the kinase enzyme in controlling the normal physiological status of a cell from the stage of embryonic development [96,98,99]. In response to ligand binding and subsequent activation of a variety of tyrosine kinase receptors, PI3K phosphorylates phosphatidyl-inositol diphosphate (PIP2) to form phosphatidyl-inositol triphosphate (PIP3) which then facilitates the phosphorylation and activation of Akt (serine/threonine specific protein kinase) through PDK1 [97,100]. Akt mediated inhibition of tuberous sclerosis complex (TSC 1 and 2) then stimulates the mTOR which, in turn, modulates its downstream effectors eIF4E binding protein 1 (4EBP1) and p70S6 kinase to modulate the synthesis of proteins that regulate cell survival, apoptosis, metabolism, proliferation, angiogenesis, glucose uptake, and ribosome biogenesis [97,100,101].

The deregulation of the PI3K/Akt/mTOR pathway plays a fundamental role in breast cancers, including cases of advanced TNBCs (Figure 1) [97,100,102,103,104,105,106]. Pharmacological suppression of PI3K/Akt/mTOR signaling showed inhibition of tumor growth in vivo [107]. Mutations of genes encoding PI3KCA (protein isoform of PI3K) and Akt1 (protein isoform of Akt) were reported in TNBCs [108]. Extensive chromosomal rearrangements due to DNA repair defects in TNBCs result in deletion of tumor suppressor protein PTEN (PI3K counteracting protein that dephosphorylates PIP3 to PIP2) [103]. Loss of critical tumor suppressor proteins, such as PTEN and inositol polyphosphate 4-phosphatase type II (INPP4B; dephosphorylates PIP2 to PIP), promotes proliferation and invasion in TNBCs [109,110]. PTPN12 phosphatase downregulates growth factor mediated receptor signaling and inhibits PI3K signaling, thereby suppressing EMT in breast cells [111]. Loss of PTPN12 in TNBCs leads to aberrant activation of the PI3K/Akt/mTOR pathway and is implicated in poor prognosis in TNC patients [111,112,113]. Kinesin family member 14 (KIF14) mediated phosphorylation of Akt promoted chemo-resistance in TNBCs [114]. The mTOR pathway is frequently activated in TNBCs compared to other subtypes of breast cancer and is correlated with poor outcome among TNBC patients [97,115,116]. Overexpression of oncogene DEK (a CAN nucleoporin fusion nuclear protein that regulates DNA damage repair, DNA replication, mRNA splicing, differentiation, and apoptosis) promoted EMT and angiogenesis via the activation of the PI3K/Akt/mTOR pathway in TNBCs [106]. Monotherapy using several different PI3K, Akt, and mTOR inhibitors or, alternatively, a combination of these inhibitors with classical chemotherapeutic agents are currently being tested in clinics for their efficacy in the treatment of TNBCs [26,97,102,103,113,117,118]. Using oral Akt inhibitor ipatasertib in combination with paclitaxel showed a longer progression-free survival in TNBC patients [119]. Additionally, crosstalk mechanisms between the PI3K/Akt/mTOR and the Ras/Raf/MEK pathways provides the venue for simultaneous targeting of both pathways to bring about a better therapeutic efficacy and outcome [100].

### 3.5. MAPK (Ras/Raf/MEK/ERK) Signaling and TNBCs

The highly conserved Ras/Raf/MEK/ERK pathway (also known as the mitogen-activated protein kinase (MAPK) cascade) transmits signals from cell surface receptors, upon ligand binding, to the nuclear transcription factors that culminate in modulation of gene expression that impacts cell survival, proliferation, motility, adhesion, angiogenesis, and invasion [120,121,122]. Knockout of MAPK pathway components, such as BRaf, MEK1, ERK1 or ERK2, during murine development lead to embryonic lethality, thereby indicating the importance of this pathway in normal physiological development [123,124,125]. Among the four MAPK pathways known to function in the humans, the one involving MEK1/2 and ERK1/2 is most relevant in carcinogenesis, especially for breast cancer [126,127]. Extracellular growth factors (ligand) bind to membrane receptors (EGFR) resulting in a conformational change that activates auto-phosphorylation of the receptors [122]. The resulting GRB2 (growth factor receptor binding protein) binding (via the SH2 domain) to the phosphorylated receptor and subsequent binding of GRB2 to SOS1 (son of sevenless 1, a guanine nucleotide exchange factor) activates the Ras protein to exchange GDP for GTP [128,129,130,131,132]. This nucleotide exchange and activation of Ras in turn activates the Raf-1 (a MAP3K) protein, which then phosphorylates mitogen-activated protein kinase ERK kinase 1/2 (MEK1/2, the MAPKK). MEK1/2 can directly mediate the phosphorylation and activation of extracellular signal-regulated kinase 1/2 (ERK1/2, the MAPK) [121,128]. The signals are then transmitted to MAPKAP (mitogen-activated protein kinase activated protein) kinase and multiple other substrates, which may be localized in the cytoplasm, nucleus, or other cellular organelles to modulate various biological responses [128,133]. Direct binding of ERK1/2 to the DNA, acting as translational co-modulators, was also reported [121,134,135].

The Ras/Raf/MEK/ERK pathway is one of the most studied kinase cascades in tumorigenesis and tumor biology, and its deregulation was frequently observed in different cancers. Therefore, targeting this pathway could be beneficial in cancer treatment. While gene mutations are relatively rare in this pathway among breast cancer patients, deregulation of this pathway at the gene expression level is a potentially significant factor in TNBCs (Figure 1) [100,136,137]. H high Ras-ERK pathway activity and higher expression of several gene sets of the Ras/Raf/MEK/ERK pathway were more common in TNBCs than other molecular subtypes of breast cancer, which suggests that inhibition of MEK signaling should be effective in the treatment of TNBCs [100,136,137,138]. In in vitro and in vivo model (mouse) TNBCs manifested a higher oncogenic Ras activity, making them more sensitive to MEK inhibition than luminal or HER2 positive subtypes [100,136]. However, therapeutic targeting of the Ras/Raf/MEK/ERK pathway alone is often less effective due to inherent cellular escape mechanisms, mainly through the activation of the PI3K pathway (and vice versa) due the significant crosstalk between the two pathways [138,139,140,141]. This indicates that while monotherapies tend to fail, combining two or more targeted agents that counteract both the PI3K and Ras-ERK pathways should be a more rational and a better approach for the treatment of TNBCs [26,142].

### 3.6. Hedgehog Signaling and TNBCs

The evolutionarily conserved, highly organized Hedgehog (Hh) signaling pathway is crucial for embryonic development, tissue/organ patterning, stem-cell renewal, cellular proliferation and differentiation, and tissue regeneration and repair [29,143]. The signaling cascade is mediated by three secreted (from the Hh producing cells) ligands, the Sonic Hh (SHh), the Indian Hh (IHh), and the Desert Hh (DHh); a transmembrane receptor-Patched (Ptch); and co-receptor Smoothened (Smo) [29,144]. Hh signaling also requires the presence of cilia (a microtubule-associated projection on the cell surface) [28]. In the absence of the Hh ligand, Ptch localizes on the cilia, interfering with Smo ciliary trafficking and activation [145]. Hh binding to the Ptch receptor leads to Ptch inactivation, which subsequently alleviates the repression on Smo, allowing its translocation into the ciliary surface, resulting in the activation and nuclear translocation of Gli (glioma-associated oncogene, zinc-finger proteins), the transcriptional mediator of Hh response [28,143,144]. Gli are bi-functional in that they can both activate (Gli1 and Gli2) and inhibit (Gli3) transcription [29,146].

Aberrant Hh signaling was reported across several cancers and plays significant roles in tumor initiation, progression, angiogenesis, EMT and metastasis, and invasion [29]. Hh activation leads to increase in the levels of angiogenic protein angiopoietin 1 (Ang-1) [147] and cell cycle proteins-cyclin D1 and B1 [148], while suppressing the levels of pro-apoptotic proteins (Fas) [29,149]. Hh mediated increase in the Snail protein and subsequent decrease in E-cadherin and tight junctions, thereby facilitating EMT in cancers [150]. Evidence suggests that overexpression of Hh signaling molecules and dysregulation of the Hh pathway confers aggressiveness on TNBCs (Figure 1) [28,151]. Smo activation leads to activation of cyclin D1 and FoxM1 proteins, which support the development and progression of TNBCs [28,29]. Additionally, overexpression of Gli1 and VEGF receptor 2 (VEGFR2) was observed in TNBCs [152]. Gli1 activation promoted TNBC progression and regulated VEGF signaling leading to tumor vascularization, while pharmacological inhibition of Smo counteracted the effects of Gli1 on tumor angiogenesis [152]. Resistance to doxorubicin, paclitaxel, and cisplatin in TNBCs was mediated by Gli1 activation and upregulation of multidrug resistance protein-1 (MDR-1) [28]. Activated Hh signaling thus endows TNBCs with enhanced self-renewal, tumor-initiating capacity, metastatic potential and invasiveness, and resistance to apoptosis and therapeutic intervention.

## 4. Phytochemicals as Anticancer Compounds Effective in the Treatment of TNBCs

**Figure 2:**
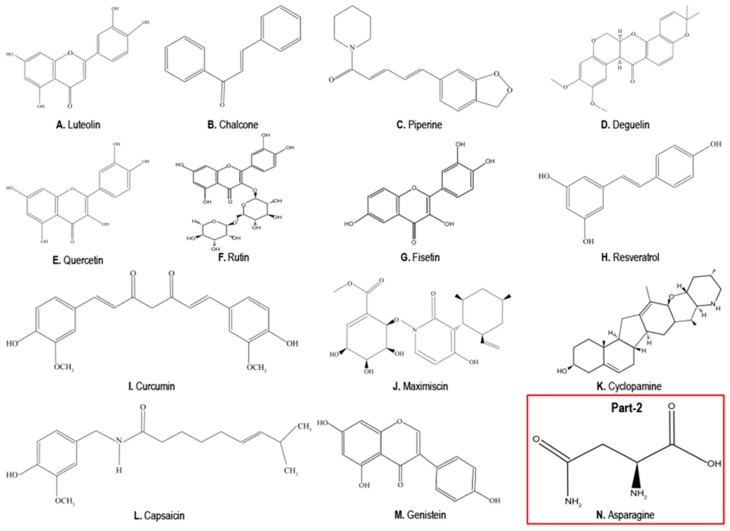
Chemical structures of the natural compounds. Image (Part 1) shows the chemical structures of the natural compounds (A. Luteolin, B. Chalcone, C. Piperine, D. Deguelin, E. Quercetin, F. Rutin, G. Fisetin, H. Resveratrol, I. Curcumin, J. Maximiscin, K. Cyclopamine, L. Capsaicin, and M. Genistein) with potential anticancer properties in TNBCs. Image Part 2 (in red box) shows the chemical structure of N. Asparagine which supports the growth and metastasis of TNBCs.

Plants are an important source of anticancer agents [153]. Consumption of many different fruits and vegetables has proved to reduce the risk of cancer, and hence in recent years much attention has been drawn towards dietary implications for cancer prevention [154]. In the last few decades, research was focusing on identifying plants (as source of the compounds) and the compounds isolated from them (phytochemicals) for their potential anticancer properties [155]. Several studies reported the scope of using plant-derived therapeutic bioactive compounds such as polyphenols, bioflavonoids, carotene, vitamins, minerals, etc. for the treatment of different cancers owing to their efficacy and lesser side effects [15,16,17,154]. Here, we highlight the potential of using phytochemicals (luteolin, chalcones, piperine, deguelin, quercetin, rutin, fisetin, resveratrol, curcumin, maximiscin, cyclopamine, capsaicin, and genistein) in the treatment of TNBCs and provide an in-depth analysis of their mechanisms of action. Figure 2 provides the chemical structures of the natural compounds which have been discussed in this article.

**Figure 3:**
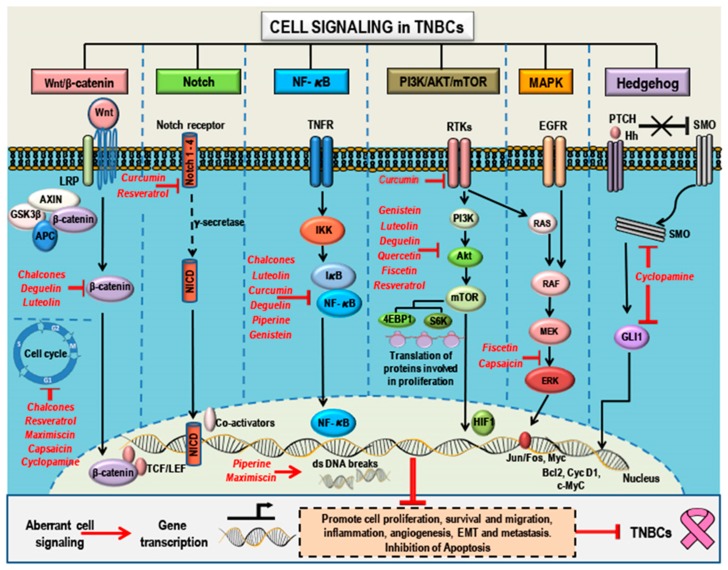
Natural compounds as anticancer agents in TNBCs: Mode(s) of Action. Aberrant signaling pathways (Wnt/β-catenin, Notch, NF-κB, PI3K/AkT/mTOR, MAPK, and Hedgehog) and pathway components that are targeted by natural compounds (highlighted in red). Phytochemicals have a broad range of action and a single anticancer phytochemical can target multiple pathways that determine cell fate. These compounds can suppress proliferation, growth, and migration; cause cell cycle arrest; induce apoptosis; inhibit angiogenesis; suppress EMT; and inhibit metastasis by targeting and modulating different pathway components and thereby regulating gene transcription and translation. This figure represents membrane, cytoplasmic; and nuclear targets of the selected natural compounds which show potential anticancer properties in TNBCs (see text for detailed mode(s) of action for the natural compounds mentioned).

**Figure 4:**
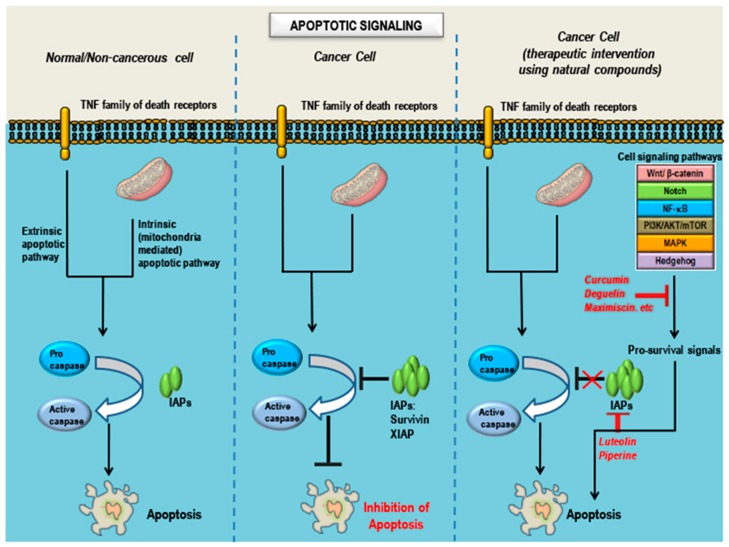
Natural compounds as pro-apoptotic agents in TNBCs: Mode(s) of Action. Apoptotic signaling in three different cell conditions. Apoptosis is regulated by several proteins including caspases, proteins that promote apoptosis such as Smac/Diablo/Bax/Bad (positive regulators), and proteins that inhibit apoptosis such as survivin/Bcl2/Bcl-xL/IAPs/XIAP (negative regulators). In a non-cancerous cell, after completion of the normal life span, the cell undergoes apoptosis/programmed cell death. Inhibition of apoptosis is a hallmark of all cancers and is characterized by abnormal expression patterns of different proteins involved in the apoptotic signaling, including an increase in levels of negative regulators, a decrease in the levels of positive regulators, and inactivation of caspases. Treatment of cancers with natural compounds/phytochemicals that possess anticancer properties can block pro-survival signals from the aberrant signaling pathways involved in oncogenesis, decrease the levels of negative regulators, increase the levels of positive regulators, and activate caspases, ultimately inducing apoptosis in cancer cells (see text for detailed mode(s) of action for the natural compounds mentioned).

These natural compounds were collected through an exhaustive search of reports and studies on the Internet and in databases. This review revealed both the in vitro and in vivo efficacy of these compounds in treatment of TNBCs and the ability of these compounds to target one or more of the aberrant/dysregulated signaling pathways in TNBCs, while maintaining diversity of compounds rather than any specific underlying physicochemical features. Figure 3 and Figure 4 summarize the signaling pathways important in TNBCs and indicate the targets and modes of action of these promising natural compounds. Treatment with these compounds leads to one or more of the effects of suppression of cell growth, proliferation, migration, inflammation, angiogenesis, EMT and metastasis (Figure 3), and induction of apoptosis (Figure 4) in TNBCs.

### 4.1. Luteolin

Luteolin (Figure 2A, Figure 3 and Figure 4) is a flavone (a type of flavonoid) traditionally used in Chinese medicine for the treatment of cancer, Alzheimer’s, multiple sclerosis (MS), rheumatoid arthritis (RA), and diabetes [156]. Luteolin possesses antioxidant [157], anti-inflammatory [158], and anticancer properties [159]. The anticancer properties of luteolin are attributed to its ability to inhibit proliferation; induce apoptosis; reverse EMT; and inhibit angiogenesis, invasion, and metastasis [159]. The cancer growth-inhibitory property of luteolin was reported in different cancer cell lines (see Table 2). It modulates cell cycle progression and its related transcription factors by targeting Vaccinia-related kinase 1 (VRK1) [160]. Luteolin binds to the catalytic site of VRK1 kinase, thus suppressing VRK1 kinase mediated BAF phosphorylation and leading to cell cycle arrest [160]. Anti-proliferative and anti-metastatic action of luteolin was shown in TNBC cell lines (see Table 2). Luteolin inhibits cell migration and viability of MDA-MB-435 and MDA-MB-231 (4175) LM2 cells [161]. Luteolin (10 and 50 µM) suppressed the secretion of VEGF, a potent angiogenic and cell survival factor, and the invasion and metastasis of MDA-MB-435 and MDA-MB-231 (4175) LM2 cell to the lungs in an in vivo mouse model of metastasis [161]. Additionally, the effect of luteolin on β-catenin (a key regulator of the Wnt signaling pathway) on p90 ribosomal S6 kinase (RSK) of the Notch signaling pathway, on PI3K/Akt pathway, and on NF-κB pathway was reported [156]. Luteolin treatment suppressed metastasis in TNBC cell lines by reversing EMT via the reduction of β-catenin expression [162]. p90 ribosomal S6 kinase (RSK), which is required for TNBC growth and survival, was identified as a potential target for luteolin [163]. Luteolin downregulated RSK1 and RSK2 kinase activity, thereby blocking Notch4 signaling, an essential for maintaining the cells in an undifferentiated state [163]. Notch signaling-related miRNAs are critical in breast cancer development and progression [164]. Luteolin inhibited Notch signaling in TNBCs by modulating the level of miRNAs (miR-181a, miR-139-5p, miR-224, and miR-246 expression levels were enhanced while miR-155 level decreased) subsequently reducing the levels of cyclin D1, Hes-1, Hey, VEGF, MMP2, and MMP9 and thereby reducing TNBC cell survival and migration [164]. Luteolin also increased the levels of anti-angiogenic miR-34a specifically in MDA-MB-231 TNBC cells leading to inhibition of cell survival, migration, and angiogenesis [164]. Luteolin also inhibited tumor cell survival, angiogenesis, and invasion in TNBC breast cancer xenografts in an in vivo mice model and was safe with no toxic/negative side effects [164]. Reports also suggest that luteolin induced cell death by suppressing PI3K/Akt, NF-κB, and X-linked inhibitor of apoptosis protein (XIAP) [156]. Furthermore, apart from luteolin monotherapy, luteolin has improved efficacy when used in combination with other drugs. Luteolin in combination with other natural compounds (epigallocatechin-3-gallate, quercetin) showed synergistic/additive growth-inhibitory effects in different cancer cell lines including TNBCs [165,166]. Luteolin in combination with tamoxifen inhibited growth in tamoxifen resistant breast cancer cells via Nrf2 downregulation [167].

### 4.2. Chalcones

Chalcones (Figure 2B and Figure 3), aromatic ketones present in a wide variety of fruits and vegetables, are known to exhibit anti-inflammatory, anticancer, anti-proliferative, antioxidant, anti-malarial, anti-bacterial, anti-fungal, and anti-viral (anti-HIV) properties [199,200]. Many chalcones were chemically modified to improve their therapeutic efficacy leading to the development of several chalcones with improved anticancer properties by targeting multiple signaling pathways [201].

Chalcones caused G2/M cell cycle arrest and were cytotoxic in MDA-MB-231 (TNBC cells) and MCF-7 cells [202]. Treatment with chalcones in MDA-MB-231 TNBC cells markedly decreased the protein levels of cyclin B1, cyclin A, and Cdc2 while significantly increasing the levels of p21 and p27 in a p53-independent manner, contributing to the G2/M cell cycle arrest [202]. A novel isochalcone, DJ52, showed a significant decrease in the proliferation of TNBC cells and a marked reduction in tumor volume in SCID mice injected with MDA-MB-231 TNBC cells [203]. TUB091 and TUB099, tubulin-binding chalcones, showed microtubule-destabilizing, vascular-targeting, antitumor, and anti-metastatic properties [204]. TUB099, a water-soluble L-Lys-L-Pro derivative of TUB091, completely abolished metastasis of MDA-MB-231/4mRL.luc2 human breast cancer cells in an in vivo mouse SCID breast cancer model [204]. A novel chalcone-based molecule, BDP, inhibited the growth of MDA-MB-231 TNBC cells by suppressing the function of Hsp90 [171]. SL4, a chalcone derivative, caused inhibition of tumor invasion and angiogenesis through suppression of HIF1 activity and activated apoptotic cell death by promoting oxidative stress [205]. SL4 was able to inhibit the proliferation of different types of breast cancer cell in vitro and in vivo by inducing G2/M cell cycle arrest [205]. The SL4 mediated inhibition of cell proliferation and induction of G2/M cell cycle arrest in TNBC cells was attributed to the SL4 related reduction in the expression of cyclin A2 and cdc25C and decrease in the activity of the cdc2/cyclin B1 complex, while increasing the levels of p21 mRNA and protein through modulation of the MAPK signaling pathways [205]. SL4 was less toxic and well tolerated in in vivo mouse models where SL4 suppressed the growth of established breast tumors in nude mice through upregulation of p21 and downregulation of cdc25C [205]. Cardamonin, a chalcone, caused downregulation of Wnt/β-catenin signaling cascades and reversal of epithelial-mesenchymal transition, thereby inhibiting TNBC invasiveness [172]. Cardamonin, 2-hydroxychalcone, and xanthohumol have growth-inhibitory effects in mesenchymal subtypes of TNBCs (BT-549, Hs578T) by modulating Bcl-2, Bax, Cyt-c, cleaved caspase-3, and PARP [172,206]. Quinolone chalcone compounds, CTR-17 and CTR-20, preferentially induced cell death in malignant multidrug-resistant breast cancer cells, including TNBCs, over non-cancerous cells [207]. CTR-17 and CTR-20, alone or in combination with paclitaxel, showed strong anticancer activity in an in vivo mouse model where the mice were engrafted with the MDA-MB-231 triple-negative breast cancer cells, without causing any notable side effects [207].

### 4.3. Piperine

Piperine (Figure 2C, Figure 3 and Figure 4), an alkaloid responsible for the spicy flavor of black and long pepper, is largely present in plants belonging to Piperaceae family. In cancer, piperine inhibits angiogenesis [208] and the tumor necrosis factor-α (TNF-α)-induced activation of NF-κB [209]. Piperine is a potent inhibitor of P-glycoprotein (P-gp) and therefore when co-administered with certain drugs (such as vincristine, colchicine, or paclitaxel) could reverse drug resistance in P-gp overexpressing cancer cells [175,210,211]. Piperine inhibits the CSC renewal through inhibition of the Wnt/β-catenin signaling pathway [175,212]. 

In TNBC cells, piperine inhibits cell proliferation, was selective for cancer cells, and did not affect the growth of normal breast epithelial cells [173]. While piperine treatment significantly decreased the levels of proteins related to the G1 and G2 phases of the cell cycle, the levels of pro-apoptotic p21 were significantly increased in piperine-treated TNBC cells [173]. Additionally, piperine inhibited the activation of pro-survival protein, Akt, and induced caspase-dependent apoptosis via the mitochondrial pathway [173]. Piperine treatment also inhibited TNBC cell migration and the expression of matrix metalloproteinase (MMP) enzymes, MMP2 and MMP9, indicative of its ability to interfere with and suppress the metastatic potential of TNBCs [173]. In immune-deficient mice, intra-tumoral administration of piperine inhibited the growth of TNBC xenografts [173]. In another study, piperine monotherapy suppressed the growth in MDA-MB-468 cells while it did not influence cell growth in MDA-MB-231 cells [174]. However, unlike growth suppression, piperine treatment did not induce apoptosis of MDA-MB-468 cells [174]. Additionally, piperine treatment caused G2/M cell cycle arrest in in MDA-MB-468 cells but not in MDA-MB-231 cells [174]. However, the combination of piperine and tumor necrosis factor-related apoptosis-inducing ligand (TRAIL) showed synergistic effects and suppressed cell growth and induce apoptosis in both TRAIL-sensitive (MDA-MB-231) and TRAIL-resistant (MDA-MB-468) human TNBC cells [174]. Piperine thus enhanced the efficacy of TRAIL-based therapies for TNBCs through the suppression of both the phosphorylation of p65 (of NF-κB pathway) and the expression of survivin (inhibitor of apoptosis) [174,213].

### 4.4. Deguelin

Deguelin (Figure 2D, Figure 3 and Figure 4), a naturally occurring rotenoid from the flavonoid family, is a natural insecticide and a fish poison [175]. The anticancer effects of deguelin were reported in various types of cancers such as prostate cancer, gastric cancer, leukemia, lung cancer, etc. [214,215,216]. Deguelin treatment selectively suppressed cell growth and induced apoptosis in non-TNBC breast cancer cells, but not in normal cells, owing to the deguelin-associated inhibition of the PI3K/Akt pathway and suppression of the expression levels of survivin and XIAP [217]. Additionally, deguelin treatment abolished drug-induced upregulation of survivin expression and sensitized breast cancer cells to docetaxel and doxorubicin treatment [217].

Deguelin treatment suppressed cell growth and proliferation in a dose-dependent manner in breast cancer cells (MCF-7, BT474, T47D, and MDA-MB-231), with the highest growth suppression being observed in MDA-MB-231 TNBC cells, which peaked at 72 h of exposure to deguelin [218]. Cell cycle analysis revealed deguelin-induced, S-phase cell cycle arrest in MDA-MB-231 TNBC cells while a G1 phase cell cycle arrest was observed in non-TNBC cells [218]. The overall deguelin treatment-associated inhibition of cell growth in MDA-MB-231 cells was due to S-phase cell cycle arrest and induction of apoptosis. Mechanistically, deguelin inhibited multiple pro-survival pathways such as Wnt/β-catenin, NF-κB, PI3K/Akt, and p38 MAPK signaling mechanisms with simultaneous induction of pro-apoptotic proteins in TNBCs, resulting in inhibition of cell survival and proliferation [218]. Deguelin treatment significantly reduced cell proliferation and growth in several different TNBC cell lines [176]. Intraperitoneal administration of deguelin was non-toxic, had no-side effects, and markedly reduced tumor growth of MDA-MB-231 cells transplanted subcutaneously in athymic mice [176]. These effects of deguelin in TNBCs were mediated through the PI3K/Akt, MAPK, and NF-κB signaling pathways by downregulating their downstream targets such as p-STAT3, c-Myc, and survivin [176]. In murine 4T1 cells (murine mammary cancer cell line developed from 6-thioguanine resistant tumor), treatment with deguelin inhibited cell growth and proliferation in time- and dose-dependent manner in conjunction with reduced nuclear PCNA staining [219]. Deguelin treatment reduced the expression of nuclear c-Met and its downstream targets such as phosphorylation of ERK and Akt [219]. Scratch wound assay revealed deguelin treatment-associated inhibition of cell migration [219]. Deguelin administration was safe/non-toxic and well tolerated and reduced the occurrence of metastatic lung lesions in an in vivo Balb/c female mice model were 4T1 cells were intravenously introduced into circulation [219].

In LAR subtype of TNBCs in MDA-MB-453 and SUM-185PE cell lines, deguelin treatment significantly reduced cell proliferation via inhibition of PI3K/Akt/mTOR signaling and decreasing androgen receptor (AR) levels and nuclear localization [220]. Deguelin mediated inhibition of phosphorylation of Akt and decrease in the expression of survivin, sensitized deguelin treated MDA-MB-231 cells to radiation and attenuated radiation induced upregulation of pro-survival Akt signaling, causing prolonged G2/M cell cycle arrest and the induction of caspase mediated apoptosis [221].

### 4.5. Quercetin

Quercetin (Figure 2E and Figure 3), a flavanol, has antioxidant, anti-inflammatory, and anticancer properties [154,168]. Quercetin-mediated growth inhibition and activation of pro-apoptotic mechanisms were observed in different cancer cell models, including breast cancers [222,223,224]. Quercetin related, suppression of cancer cell proliferation operates via inhibition of intracellular signaling pathways such as PI3K, EGFR and Her2/neu, while the induction of cancer cell apoptosis is modulated via inhibition of the survival signaling pathways (Akt, NF-κB) or regulatory molecules associated with cell apoptosis (p53, Bcl-2 family, FasL) [222]. Quercetin treatment induces downregulation of anti-apoptotic Bcl-2 and upregulation of pro-apoptotic Bax, increases the levels of cytochrome C levels and cleaved forms of caspase-9, caspase-3 and PARP-1 [225].

Quercetin-mediated activation of caspase-3, -8 and -9, increase in levels pro-apoptotic protein Bax, decrease in levels of anti-apoptotic protein Bcl-2 and release of apoptosis-inducing factor (AIF) from mitochondria and its subsequent translocation into the nucleus correlated with the dose- and time-dependent G2/M cell cycle arrest and apoptosis (more cells in the sub-G1 phase) in quercetin treated MDA-MB-231 TNBC cells [226]. Reportedly, the anti-proliferative effects of quercetin through G2/M cell cycle arrest and apoptosis in human luminal androgen receptor-positive MDA-MB-453 TNBC cells is mediated through increased Bax expression; cleaved caspase 3 and PAR expression; and decreased Bcl-2 expression [227]. In a C3(1)/SV40 Tag breast cancer mouse model, quercetin administration was well tolerated and reduced the number of tumor cells and their volume in a dose-dependent manner [228].

In MDA-MB-231 cells, quercetin treatment caused a JNK pathway-mediated increase in the protein level, transcriptional activity, and nuclear translocation of pro-apoptotic Foxo3a, which in turn activated cell death mechanisms via p53, p21, and GADD45 signaling [222]. Additionally, quercetin treatment also increased the expression of Fas ligand (FasL, a pro-apoptotic factor that activates Fas receptor-mediated apoptosis) mRNA [222]. These quercetin treatment-associated pro-apoptotic events reduced viability, induced apoptosis, and caused S-phase and G2/M cell cycle arrest, along with a reduction in cells in the G0/G1 phase of the cell cycle [222].

Quercetin treatment was associated with a significant growth-inhibitory effect in MDA-MB-231 and MDA-MB-157 TNBC cells [177]. Quercetin treatment in these TNBC cells induced morphological alterations, DNA fragmentation, and caspase-3 activation, in addition to decreased protein expression of lipogenic enzyme FasN, anti-apoptotic Bcl-2, β-catenin, and reduced nuclear accumulation of β-catenin, indicating a quercetin-mediated inhibition of the Wnt/β-catenin signaling in TNBCs [177]. Quercetin administration also significantly decreased MDA-MB-231 breast tumor xenograft growth in mice [177]. Quercetin treatment-associated reduction in nuclear translocation of β-catenin and subsequent downregulation of Wnt/β-catenin signaling-related genes such as cyclin D1, c-Myc at the mRNA and protein level led to a decrease in growth, survival, migration, and altered morphology in MDA-MB-231 and MDA-MB-468 TNBC cells [229]. Quercetin treatment-associated decrease in the phosphorylation of Akt could have contributed to the decrease in the phosphorylation of GSK-3β, thereby indicating that quercetin can target multiple signaling pathways simultaneously [229]. Furthermore, quercetin treatment decreased the levels of vimentin (increased levels support EMT) and increased the levels of E-cadherin (decreased levels supports EMT) indicating that quercetin can inhibit EMT and metastasis [229].

Quercetin treatment sensitized recombinant human TRAIL (rhTRAIL) resistant BT-20 TNBC cells to rhTRAIL induced apoptosis [230]. Quercetin treatment remodels the tumor microenvironment and thus improves the permeation, retention, and antitumor effects of nanoparticles [231]. A nano-carrier-mediated delivery of a combination of topotecan and quercetin facilitated selective uptake of the nanoparticle containing the drug combinations in MDA-MB 231 cells through integrin receptor-mediated endocytosis, followed by intracellular dissociation of the nanoparticle, releasing the drugs which in turn subsequently triggered TNBC cell death [232]. In comparison to drug monotherapy, quercetin co-administration with chemotherapeutic drugs such as cisplatin [233,234,235] and plant-derived compounds (such as resveratrol and curcumin) [236,237] showed improved efficacy in the treatment of different cancers, highlighting the possibility of using quercetin-chemotherapeutic drug/plant derivative combinations in the treatment of TNBCs.

### 4.6. Rutin

Rutin (Figure 2F and Figure 3) (rutoside, quercetin-3-O-rutinoside and sophorin), a glycoside combining the flavanol, quercetin, and the disaccharide, rutinose, is widely present in citrus fruits [178,238]. Rutin has anti-carcinogenic, anti-inflammatory, and antioxidant properties [178]. In vitro studies in different cancer cells indicate that rutin exerts its anticancer potential through modulation of multiple oncogenic pathways such as the Wnt/β-catenin, NF-κB, MAPK, and PI3K/Akt signaling mechanisms; through activation of apoptosis; and by inducing DNA damage [239]. Rutin-associated inhibition of proliferation; attenuation of superoxide production; and reduction in adhesion and migration of cancer cells are reported [240]. In neuroblastoma cells rutin exerts its antitumor effect through induction of G2/M cell cycle arrest and promoting apoptotic cell death. These effects were mediated by decreased Bcl2 protein expression and Bcl2/Bax ratio, while also inhibiting the MycN mRNA level and the secretion of TNF-α [241].

Rutin treatment-associated decrease in c-Met kinase inhibited cell proliferation, migration, and invasion of MDA-MB 231 TNBC cells [178]. The receptor tyrosine kinase c-Met is dysregulated in TNBCs and contributes to TNBC progression, motility, and survival [178]. Intraperitoneal administration of rutin over a three-week treatment period reduced the growth of TNBC MDA-MB-231 xenografts in an in vivo nude mouse model of TNBC [178]. Rutin treatment-associated inhibition of P-gp transport function and significant reduction in resistance to paclitaxel in P-gp overexpressing MDR cell lines is indicative of rutin’s possible ability to sensitize chemo-resistant TNBC cells [238]. In MDA-MB-231 TNBC cells, rutin co-treatment enhanced anticancer activity and cytotoxicity of cyclophosphamide and methotrexate while decreasing the levels of -glycoprotein (P-gp) and breast cancer resistance protein (BCRP). Cell cycle analysis revealed rutin mediated G2/M and G0/G1 cell cycle arrest while significantly promoting apoptotic cell death in TNBC cells [242].

### 4.7. Fisetin

Fisetin (Figure 2G and Figure 3), a plant polyphenol from the flavonoid group, has anticancer properties [243]. The mTOR signaling, PI3K/Akt signaling, NF-κB signaling, CDK5 signaling, NRF2-mediated oxidative stress response, glucocorticoid signaling, and MAPK signaling are the key signaling pathways modulating the growth-inhibitory effects of fisetin in different cancers, including breast cancers [243,244]. Fisetin treatment triggered the expression of pro-apoptotic proteins such as Bad, Bak, Bid, and Bax; decreased the expression of anti-apoptotic proteins Bcl2 and Bcl-xL; activated caspases; and induced PARP cleavage [243]. Fisetin-loaded folate-functionalized pluronic micelles can overcome fisetin’s poor aqueous solubility and bioavailability in an in vivo setting, thereby improving the efficacy of drug delivery as well as the anticancer effect of fisetin in breast cancer [245].

Fisetin exposure inhibited cell growth and colony forming ability in MDA-MB-468 and MDA-MB-231 TNBC cells without significantly affecting the growth of non-malignant cells [246]. The fisetin-mediated inhibition of TNBC cell proliferation and induction of apoptosis is related to mitochondrial membrane permeabilization and the activation of caspase-9 and caspase-8 PARP1 cleavage [246]. Cell cycle analysis revealed fisetin induced G2/M cell cycle arrest. Fisetin treatment triggered apoptosis due to the inhibition of Aurora B kinase, a protein required for the attachment of spindle fiber to centromere (regulates chromosome segregation) during mitosis and meiosis [246]. Additionally, fisetin exposure increased the sensitivity of TNBC cells to cytotoxic effects of cisplatin, 5-fluorouracil, and the 4-hydroxycyclophosphamide metabolite of cyclophosphamide [246].

### 4.8. Resveratrol

Resveratrol (Figure 2H and Figure 3), trans-3,4′,5-trihydroxystilbene, is a natural non-flavonoid polyphenolic compound. Resveratrol, its analogs, and its metabolites are extensively studied in a large spectrum of pathological conditions, and they confer protection against metabolic, cardiovascular, and age-related diseases such as neurodegeneration and cancer [18,247,248]. Several clinical trials are currently underway to investigate the effect of resveratrol in different diseases, including cancers [18,21,249,250].

The molecular targets of resveratrol include AMPK; silent information regulator-2 (Sir2 in yeast, Sirtuin1/Sirt1 in mammalian system); cyclooxygenases (COX); phosphodiesterases; and components of the NF-κB, PI3K/Akt/mTOR, MAPK and Hedgehog signaling pathways [247,248,250,251]. Resveratrol exerts its anticancer effects in different cancers by reducing ROS, COX, and cytokine expression (antioxidant and anti-inflammatory effect); by decreasing the activity of transcription factors such as NF-κB and AP-1 (anti-proliferative and anti-survival effect); by decreasing cyclins and CDKs (anti-proliferative effect and inhibition of cell cycle); by increasing pro-apoptotic p53 and Bax while decreasing Bcl2 and survivin (pro-apoptotic effect); and by decreasing HIF1α and MMPs (anti-angiogenic and anti-migratory effect) [247,252]. In different breast cancers (in vitro), resveratrol treatment suppressed tumor cell proliferation, growth, and migration; inhibited angiogenesis; caused cell cycle arrest; induced apoptosis; activated pro-apoptotic pathways while suppressing anti-apoptotic pathways; inhibited EMT; quenched oxidative radicals; sensitized tumor cells to chemotherapeutic agents; and suppressed multidrug resistance in breast cancer cells [248,253,254]. In in vivo mice models of breast cancer, resveratrol treatment had no adverse side effects; was relatively safe; suppressed mammary gland differentiation, proliferation, lipid peroxidation, angiogenesis, migration, and metastasis; reduced tumor volume; and induced apoptosis [248,253].

In MDA-MB-231 TNBC cells, treatment with resveratrol inhibited growth and induced PARP1 cleavage-mediated apoptosis [181]. Mechanistically, resveratrol treatment caused transient activation of p44/42 MAPK while inhibiting the phosphorylation and activation of S6 ribosomal protein (which plays an important role in protein translation) [181]. The inhibition of cell growth in response to resveratrol treatment was abolished in the presence of MAPK, suggesting that these effects of resveratrol are dependent on MAPK signaling [181]. The data demonstrated that resveratrol-induced apoptosis is associated with MAPK signaling and with the inhibition of proteins that are involved in protein translation [181]. Resveratrol administration significantly reduced tumor growth, decreased angiogenesis, and increased apoptosis in MDA-MB-231 tumor xenografts in a resveratrol-treated nude mouse model of TNBC [182]. A significant decrease in apoptosis in addition to significantly reduced extracellular levels of VEGF was observed in resveratrol-treated MDA-MB-231 TNBC cells [182]. A combination of rapamycin and resveratrol decreased cell growth and inhibited mTOR signaling in MDA-MB-231 cells, while preventing Akt activation and autophagy, thus causing apoptosis [255]. Additionally, resveratrol treatment-mediated downregulation of the MDR1 and CYP2C8 genes (which confers resistance to paclitaxel in resistant TNBC cells) reduced cell proliferation and colony formation and increased senescence and apoptosis in paclitaxel-resistant MDA-MB-231 cells [256]. Several studies in mouse and human TNBC cell lines shed light on the potential of using resveratrol in the treatment of TNBCs [253]. The anticancer effects of resveratrol in TNBCs—such as suppression of growth, proliferation, and migration; induction of apoptosis; reduction in drug resistance; increased sensitivity to cytotoxic drugs; and inhibition of metastasis and invasion—are mediated through targeting multiple signaling pathways and modulation of several target genes and proteins [253,257,258,259,260,261,262]. Several studies documented suppression of tumor growth as well as reduced angiogenesis, metastasis, and invasion in resveratrol-treated in vivo TNBC mouse xenograft models through the modulation of several different signaling pathways and their target genes [182,253,257,260,263]. Resveratrol in combination with other chemotherapeutic drugs (rapamycin, tamoxifen, doxorubicin, paclitaxel, cyclophosphamide, etc.) or other anti-cancerous phytochemicals (pterostilbene, quercetin, curcumin, genistein, EGCG, catechin etc.) improved the efficacy of the treatment in TNBCs [253,255,264,265,266,267,268].

Other studies reported possible adverse effects of resveratrol treatment in breast cancers. Resveratrol treatment caused HER2 accumulation and preferential activation of the mTOR pathways, thereby supporting HER2+/ERα+ breast cancer growth [269]. In TNBC cells, resveratrol exposure reduced susceptibility to paclitaxel-induced cell death due to the resveratrol-mediated inhibition of paclitaxel-induced G2/M arrest, together with elevation in the levels of cyclin B/CDK1 and suppression of paclitaxel-induced oxidative stress [270,271,272]. Concentration-dependent biphasic effects of resveratrol in different human cells were reported [273,274]. Resveratrol thus should have a dose-dependent biphasic hormetic effect in breast cancer cells [275].

### 4.9. Curcumin

Curcumin (Figure 2I, Figure 3 and Figure 4), the principal curcuminoid of turmeric (*Curcuma longa*), a member of the ginger family, *Zingiberaceae*, is a spice commonly found in the Indian subcontinent [276]. Its medicinal properties, both preventive and as a therapeutic agent for many ailments, are well documented in the ancient manuscripts of the traditional Indian medicine of Ayurveda [276]. It is used in diseases such as skin disorders, Alzheimer’s, gastro-intestinal ailments, cardiovascular diseases, arthritis, and cancer [276,277].

In cancers, including breast cancers, curcumin exerts its anticancer action by targeting multiple pathways (Wnt/β-catenin, Notch, NF-κB, PI3K/Akt/mTOR, MAPK and Hh pathways) and modulates several target genes and proteins [278]. Curcumin suppresses cell growth, proliferation, and migration,; induces apoptosis; and inhibits angiogenesis, metastasis and invasion in cancers through the modulation of several molecular targets that include transcription factors (NF-κB, AP1, HIF1, Nrf2, STATs, PPAR-γ etc.), growth factors (VEGF, FGF, HGF, PDGF etc.), inflammatory cytokines (interleukins, TNF-α, MCP etc.), kinases (PKA, PKB, MAPK, FAK, ERK etc.), receptors (FasR, ER-α, HER2, CXCR4 etc.), enzymes (ATPase, COX2, LOX-5, telomerase, MMPs etc.), and other effector molecules such as Bcl2, Bcl-xL, cyclin D1, IAPs, and p53 [279,280]. While there is a large body of scientific literature revealing the anticancer of curcumin in different cancers, we focus on studies pertaining to the use of curcumin in the treatment of breast cancers, specifically TNBCs.

In MDA-MB-231 TNBC cells, curcumin treatment significantly reduced cell proliferation and induced apoptosis through the modulation of the EGFR-MAPK signaling, as evidenced by curcumin treatment-associated decrease in the levels of phosphorylated ERK1/2 and EGFR [184]. Curcumin exposure downregulated β-catenin, Slug, AXL, and vimentin expression and upregulated E-cadherin and N-cadherin, which correlated with reduced migration and invasiveness of MDA-MB-231 cells [281]. Curcumin treatment limited the metastatic potential of MDA-MB-231 and MDA-MB-468 cells by altering the expression of cell adhesion molecules [282]. Curcumin also blocked fatty acid synthase activity and expression; increased levels of Bax (pro-apoptotic); and decreased phosphorylation of Akt and levels of Bcl2 (anti-apoptotic), thereby mediating the decrease in cell proliferation and induction of apoptosis in MDA-MB-231 cells [283]. Curcumin inhibited cell proliferation and migration and caused G2/M cell cycle arrest and induced apoptosis in MDA-MB-231 cells by increasing Bax and p21 expression, decreasing p53 and NF-κB p65 expression, and increasing the Bax/Bcl-2 ratio [284]. Various mechanisms, including modulation of Wnt/-catenin signaling, the MAPK pathway, and NF-κB signaling were reported to explain the anti-proliferative and cell death-inducing effects of curcumin in TNBCs [285,286,287,288].

Several curcumin combination drugs showed improved efficacy in the treatment of TNBCs. Curcumin treatment-related inhibition of NF-κB was linked to the chemo-sensitization of MDA-MB-231 cells to 5-fluorouracil [289]. Curcumin treatment in retinoic acid resistant TNBC cells sensitized these cells to retinoic acid-mediated growth suppression and decreased BrDu incorporation [290]. This effect of curcumin was attributed to the curcumin treatment-mediated inhibition of p65-regulated transcription and subsequent suppression of fatty acid-binding protein 5 (FABP5) [290]. The curcumin/retinoic acid combination also inhibited peroxisome proliferator-activated receptor β/δ (PPARβ/δ) target genes VEGF-A and PDK1 [290]. Interestingly, a combination of curcumin and anti-diabetic drug metformin targets breast cancer in mice by the induction of p53-independent apoptosis and inhibition of angiogenesis [291]. Combinations of curcumin and other phytochemicals such as resveratrol and quercetin were more effective than using only one of these compounds, due to the ability of combinations to bring about synergistic effects and inhibit multiple signaling pathways in TNBCs.

Despite its efficacy and safety in in vitro and in vivo models, curcumin treatment is less effective in human cancer subjects mainly due to its reduced bioavailability and rapid metabolism. Curcumin analogs with improved bioavailability and tumor retention showed improved efficacy in the treatment of TNBCs [292,293]. The reduced bioavailability of curcumin was overcome by facilitating in vivo delivery of curcumin through novel curcumin-loaded phosphorylated calixarene POCA4C6 micelles [294]. In TNBC cells, the micelles efficiently caused G2/M cell cycle arrest and inhibited proliferation, invasion, migration, and tumor spheroid formation, with associated induction of apoptosis and reduction in the levels of AR and nuclear β-catenin [294]. The curcumin-loaded micelles had improved retention in TNBC tumor xenografts of BalB/c nude mice and inhibited tumor growth without any systemic toxicity [294]. A biomimetic nanodrug consisting of a self-assembling variant of human apoferritin loaded with curcumin (CFn) improved solubility, chemical stability, and bioavailability of curcumin [295]. CFn was more effective in reducing cell viability of MDA-MB-468 and MDA-MB-231 cells than curcumin alone [295]. CFn sensitized the TNBC cells and enhanced the cytotoxic effects of doxorubicin by interfering with the activity of multidrug resistance (MDR) transporters [295]. While CFn caused G0/G1 cell cycle arrest in MDA-MB-231 cells, MDA-MB-468 accumulated in G2/M phase [295]. Mechanistically, CFn operated through inhibition of the PI3K/Akt pathway, as evidenced by a decrease in the levels of phosphorylated Akt in CFn treated TNBC cells [295]. Ultra-microsecond pulsed curcumin, curcumin-derivative nano-micelles, and curcumin-loaded nanoparticles increased the bioavailability of curcumin and was more effective than regular curcumin in the treatment of TNBCs [296,297].

### 4.10. Maximiscin

Maximiscin (Figure 2J, Figure 3 and Figure 4), a fungal metabolite isolated from fungus *Tolypocladium* sp., was discovered as part of a crowdsourcing initiative in the USA [298]. Maximiscin treatment showed growth suppression and cytotoxic efficacy towards basal-like 1, MDA-MB-468 TNBC cells when compared to other molecular subtypes of TNBCs [186]. Maximiscin administration also suppressed tumor growth in MDA-MB-468 TNBC xenografts in nude mice [186]. Mechanistically, maximiscin caused accumulation of cells in the G1-phase of the cell cycle, suggesting induction of DNA damage (double stranded breaks) leading to apoptosis with subsequent activation of DNA repair mechanisms, as evidenced by the phosphorylation and activation of p53 and check point kinases Chk1 and Chk2 [186]. Maximiscin induces growth inhibition primarily via DNA damage as indicated by high expression of cell cycle and DNA damage response proteins, suggestive of a mechanism similar to enhanced sensitivity of BL subtype to platinum-based compounds [186]. Maximiscin also circumvented P-glycoprotein (P-gp)-mediated multidrug resistance in TNBCs [299].

### 4.11. Cyclopamine

Cyclopamine (Figure 2K and Figure 3), a steroidal alkaloid isolated from corn lily (*Veratrum californicum*), a plant native to Western North America, has both teratogenic and anticancer properties [300]. Cyclopamine specifically inhibited the Hedgehog pathway during the developmental stage, and hence the offspring of sheep grazing on corn lily showed teratogenic effects with severe cranio-facial birth conditions (cyclops lamb) [300].

Impaired and activated Hedgehog signaling is implicated in many cancers, including breast cancer and specifically TNBCs [151,301,302]. Immuno-histochemical analysis of breast cancer patient tissue section samples showed significant staining for the Hh pathway proteins, smoothened (Smo), and Gli1 in TNBCs when compared to non-TNBCs [151]. Cyclopamine directly binds to and inhibits Smo protein in Hedgehog signaling, thereby blocking the Gli1-mediated modulation of genes involved in cell proliferation and survival, EMT, invasion, migration, and angiogenesis; osteolytic metastases; and chemotherapeutic resistance [28,303]. However, Smo-independent effects of cyclopamine on the growth of breast cancer cells were also reported [304].

In MDA-MB-231 TNBC cells, a marked increase in the levels of the activated Sonic Hh (SHh), Ptch, Smo and Gli1 resulted in overexpression of Bcl2 and cyclin D1, thereby contributing to cell proliferation and survival [305]. Cyclopamine treatment in these cells resulted in a decrease in Gli mRNA and cell viability which correlated with the cyclopamine treatment-associated decrease in Bcl2 and cyclin D1 [305]. Additionally, exposure of MDA-MB-231 cells to human SHh significantly reduced the levels of E-cadherin, increased MMP2 and MMP9, and enhanced cell migration and invasion, thereby contributing to EMT. This effect was reversed, and levels of E-cadherin were enhanced, while the levels of MMP2 and MMP9 decreased in cyclopamine treated cells, with a consequent decrease in cell migration and invasion [305]. Cyclopamine treatment showed significant suppression of proliferation in MCF-7 and MDA-MB-231 breast cancer cells, caused by a robust G1 cell cycle arrest and inhibition of MAPK/ERK signaling which contributed to the decrease in the expression of cyclin D1 [188]. Cyclopamine also inhibited the invasiveness in MCF-7 and MDA-MB-231 cells, as evidenced by the suppression of levels of NF-κB, MMP2, and MMP9 proteins [188]. Additionally, reports show that cyclopamine reduced viability and increased apoptotic cell death in breast cancer epithelial cell lines such as MDA-MB-435, T47D, MDA-MB-231, and MCF7 cells [306]. In MDA-MB-435 and MCF10AT cells, cyclopamine reduced transcription of Gli1, but not transcription of Ptch1, and inhibited Gli-mediated transcriptional activity [306]. Cyclopamine sensitized MDA-MB-231 cells to paclitaxel, enhanced paclitaxel-reduced cell viability, and induced cell death [307]. Additionally, co-administration of cyclopamine and paclitaxel-reduced tumor growth in MDA-MB-231 tumor xenografts in nude mice [307]. Combinations of cyclopamine and EGFR inhibitors (afatinib and gefitinib) or tamoxifen showed synergistic and improved anticancer effects in both MCF cells and MDA-MB-231 cells when compared to control cells exposed to a single drug [308,309].

Many synthetic analogs of cyclopamine (D-homocyclopamine, cyclopamine-4-en-3-one, 3-keto-*N*-aminoethyl aminocaproyl digyrocinnamoyl (KAAD)-cyclopamine) with better solubility and stability have been synthesized and tested for their anticancer properties [310]. Cyclopamine, being specific in its action of targeting the deregulated Hh signaling, stands as a promising drug candidate for the treatment of TNBCs; however, proper assessment for its toxicity and safety is advised.

### 4.12. Capsaicin

Capsaicin (Figure 2L and Figure 3) (trans-8-methyl-*N*-vanillyl-6-nonenamide) is the active component in chili pepper that gives pepper its characteristic pungent flavor [311]. Capsaicin alters the expression of several genes involved in cancer cell survival, growth arrest, angiogenesis, and metastasis by targeting multiple signaling pathways (Wnt/β-catenin signaling, PI3K/Akt/mTOR signaling, Notch signaling, endoplasmic reticulum stress signaling, MAPK signaling, etc.); oncogenes; and tumor suppressor genes in various models of cancer [312,313]. However, studies provide conflicting reports suggesting that capsaicin can act as a carcinogen or as a cancer preventive/therapeutic agent [311,314]. Capsaicin showed co-carcinogenic effects through ERK, p38 MAPK, and EGFR-dependent mechanisms in 12-O-tetradecanoylphorbol-13-acetate-induced skin carcinogenesis [315,316]. Although adverse effects of capsaicin in breast cancers have not been reported, the use of capsaicin should be cautiously considered as an anticancer agent.

In MCF7 and MDA-MB-231 cells, tumor cell growth inhibition and induction of apoptosis were correlated with levels of capsaicin present in whole hot peppers extracts [317]. The pepper extracts had little effect on non-tumorigenic breast epithelial MCF10A cells, indicating that pepper extracts are capable of selectively targeting cancer cells [317]. Exposure to purified capsaicin resulted in inhibition of cell growth in MCF7 and MDA-MB-231 cells, indicating that the capsaicin present in the pepper extracts was responsible for similar effects in breast cancer cells [317]. Additionally, treatment of MDA-MB-231 cells with hot peppers extracts showed VEGF inhibition in MDA-MB-231 cells, indicating capsaicin’s ability to inhibit metastasis and improve prognosis for cancer survival [317]. Treatment with purified capsaicin inhibited VEGF expression in mouse and human cells and suppressed tumor angiogenesis both in vitro and in vivo [317,318]. In BT20 TNBC cells, treatment with capsaicin decreased viability in a dose-dependent manner through the induction of apoptosis and causing S-phase cell cycle arrest [319]. Capsaicin treatment-related decrease in mitochondria membrane potential and cleavage of PARP-1 was observed in capsaicin-treated TNBC cells [319]. In addition to the capsaicin-induced effects observed in BT20 cells, AIF was distinctly released from mitochondria in MCF-7 cells (but not in BT 20 cells) [319]. In SUM149PT TNBC cells, capsaicin acts as an agonist for transient receptor potential V1 (TRPV1) channels, thereby activating TRPV1 in these cells and leading to an increase in TRPV1-mediated intracellular calcium signals [320]. Exposure to capsazepin, a specific TRPV1 antagonist, diminished these effects of capsaicin treatment [320]. The capsaicin-mediated activation of TRPV1 caused significant inhibition of cancer cell growth and induced apoptosis and necrosis [320]. Capsaicin significantly reduced migration and invasion of MDA-MB-231 cells and downregulated the mRNA and protein levels of MMP2 and MMP9 in a dose-dependent manner [190]. Capsaicin inhibited growth of MDA-MB-231 TNBC cells lines, which was associated with G0/G1 cell-cycle arrest, increased levels of apoptosis, and reduced protein expression of human epidermal growth factor receptor (EGFR), active extracellular-regulated kinase (ERK), and cyclin D1 [191]. Capsaicin did not have a significant effect on the proliferation of untransformed human mammary epithelial cells, MCF-10A [191]. In contrast, capsaicin treatment increased the levels of p27^KIP1^ (cell-cycle regulator) and increased caspase activity and PARP cleavage [191]. Capsaicin also blocked breast cancer cell migration in vitro and reduced the size (by 50%) of MDA-MB231 breast cancer tumors grown ortho-topically in immune-deficient mice without noticeable drug side effects [191]. The levels of activated ERK and cyclin D1 markedly decreased while caspase activity and PARP cleavage significantly increased in the tumors of capsaicin-treated mice [191].

RPF151, a novel capsaicin-like analog with improved stability, better aqueous solubility, enhanced cytotoxicity, and less pungency than capsaicin, downregulated p21 and cyclins A, D1, and D3 in MDA-MB-231 cells, leading to an S-phase cell cycle arrest and apoptosis [321]. Normal MCF10A cells were more resistant to RPF151 treatment [321]. Although RPF151 has induced the activation of TRPV1 in MDA-MB-231 TNBC cells, it’s in vivo antitumor activity in MDA-MB-231 tumors was independent of TRPV1 activation [321]. Capsaicin had synergistic anticancer activity in combination with genistein through modulation of AMPK and COX2 in MCF7 cells, indicating that using capsaicin in combination with other phytochemicals or chemotherapeutic drugs may improve the efficacy of therapeutic intervention in TNBCs [322].

### 4.13. Genistein

Genistein (Figure 2M and Figure 3), an isoflavone found in soybeans, fava beans, and coffee, is a phytoestrogen with antioxidant, anti-angiogenic, and anticancer properties [323,324]. In different cancers, including breast cancers, genistein inhibits cell proliferation and growth, causes cell cycle arrest, induces apoptosis, and modulates the NF-κB, PI3K/Akt/mTOR, and MAPK pathways [323,324,325,326,327].

In MDA-MB-231 TNBC cells, genistein treatment markedly inhibited cell growth and induced apoptosis in a dose-dependent and time-dependent manner [328]. Genistein caused accumulation of cells in G2/M phase of the cell cycle [328]. Mechanistically, the effects of genistein in MDA-MB-231 cells was related to inhibition of the NF-κB activity via the Notch-1 signaling pathway in a dose-dependent manner [328]. Additionally, genistein treatment caused NF-κB inhibition-related downregulation of the expression of cyclin B1, Bcl-2, and Bcl-xL [328]. A quantitative phosphor-proteomic approach revealed that genistein modulates cell cycle and DNA damage response pathways in TNBC cells [329]. Genistein regulated 332 phosphorylation sites on 226 different proteins, thereby modulating key processes during the cell cycle, such as DNA replication, cohesin complex cleavage, and kinetochore formation [329]. Furthermore, genistein activates DNA damage response, including activation of ATR and BRCA1 complex in TNBC cells [329]. Genistein treatment-related upregulation of Bax and p21^WAF1^ expression and downregulation of Bcl-2 and p53 expression were reported in MDA-MB-231 cells [194]. Genistein treatment also mediated PARP cleavage and induced apoptosis [194]. Exposure to genistein suppressed growth in MDA-MB-231 cells, along with G2/M cell cycle arrest and increase apoptotic cells, as evidenced by an increase in the number of cells in the sub-G0/G1 phase [330]. Western blot analysis revealed a significant increase in the levels of active phosphorylated-p53 (no change in total p53) with a subsequent increase in the levels of p21 (a p53 target gene) and marked decrease in either Bcl-xL or cyclin B1 [330]. The protein levels of PTEN, cyclin D1, and p27 remain unchanged in genistein-treated MDA-MB-231 cells [330]. However, similar effects were reported in normal MCF-10A cells, indicating that the anticancer effects of genistein may not be selective of breast cancer cells [330].

The genistein-mediated modulation of the MAPK pathway has potential anticancer effects in TNBCs [331]. Data shows that genistein treatment caused a concentration-dependent suppression of the protein levels of MEK5, total ERK5, and phospho-ERK5, and a subsequent decrease in NF-κB/p65 protein levels and the DNA-binding activity of NF-κB, consistent with the inhibition of cell growth and induction of apoptosis [331]. Genistein lowered the levels of anti-apoptotic Bcl-2, increased the levels of pro-apoptotic Bax, and resulted in cleavage and induction of caspase-3 activity in a concentration-dependent manner [331]. Genistein administration-mediated inhibition of tumor growth and invasion of breast carcinoma cells were observed in nude mice bearing MCF-7 and MDA-MB-231 xenografts, accompanied by downregulation (a gene involved in tumor cell migration) [193,332]. Genistein sensitized large cell lymphoma to chemotherapeutic agents such as cyclophosphamide, doxorubicin, vincristine, and prednisone [333]. Additionally, co-administration of genistein with drugs such as methotrexate, eicosapentaenoic acid, topotecan, centchroman, and other phytochemicals such as capsaicin showed synergistic effects in TNBC cells and other cancers, indicative of better efficacy in targeting multiple oncogenic pathways using drug combinations when compared to drug monotherapy [322,334,335,336,337].

Studies also reported a biphasic hormetic response in cancer cells (including breast cancers) for genistein. In T47D cells, treatment with low concentrations of genistein promoted cell growth by 135%, while a higher concentration of genistein (25–100 µM) induced 20–40% cell death [338]. Additionally, the estrogenic effects of genistein may contribute to its potential pro-carcinogenic property in breast cancers [339].

## 5. Phytochemicals that Can Promote TNBC Growth, EMT and Metastasis

### Asparagine

**Figure 5:**
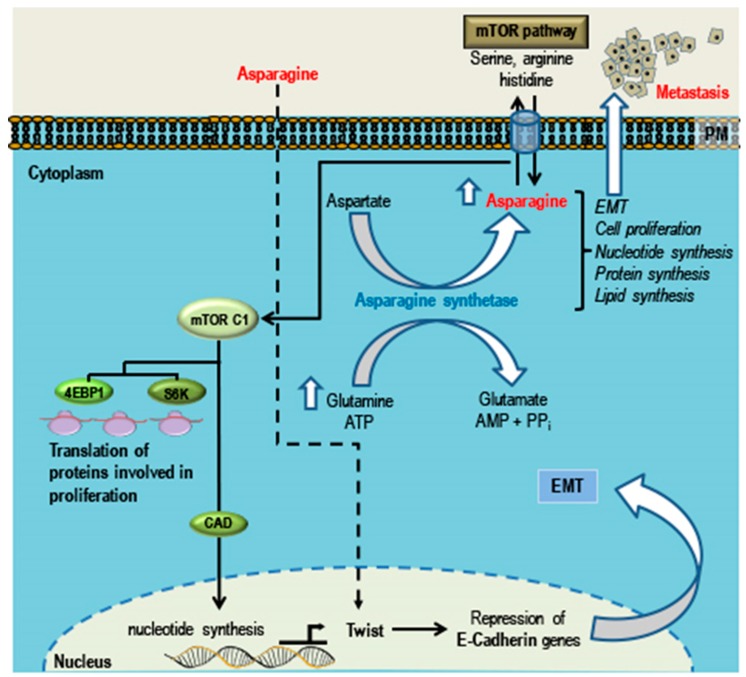
Role of asparagine in promoting growth, EMT, and metastasis in TNBCs. Asparagine, a non-essential amino acid, is synthesized in the body from aspartate in the presence of asparagine synthetase. Asparagine can act as an amino acid exchange factor regulating the uptake of other amino acids such as arginine, histidine, and serine into the cell. This amino acid exchange increases protein and nucleotide synthesis through the activation of mTORC1, which in turn favors and promotes TNBC cell proliferation. Increase in the bioavailability of asparagine (from dietary asparagine and the asparagine synthesized by the body) leads to an upregulation of EMT-up genes and asparagine-enriched EMT proteins (such as Twist1) with a decrease in the levels of E-cadherin, thereby promoting epithelial-mesenchymal transition (EMT) and supporting TNBC metastasis.

Asparagine (Figure 2N and Figure 5), a non-essential amino acid and key component in asparagus, is found in fruits, vegetables, meat, and dairy products. In the body, asparagine synthetase converts aspartate to asparagine in the presence of glutamine and ATP [340]. Figure 5 depicts the role of asparagine in promoting growth, EMT, and metastasis in TNBCs. Asparagine is important for protein synthesis and acts as an amino acid exchange factor regulating the uptake of other amino acids such as arginine, histidine, and serine (Figure 5) [340,341]. This amino acid exchange increases protein and nucleotide synthesis through the activation of mTORC1, which in turn favors and promotes cell proliferation [340,341].

In cancers, glutamine starvation-induced apoptosis is suppressed by asparagine, which inhibits endoplasmic reticulum stress and translation dependent apoptosis [342]. L-asparaginase treatment significantly induced apoptosis in cancer cells due to the depletion of asparagine [343].

Murine 4T1-T cells with a strong propensity to form circulating tumor cells require vascular mimicry to metastasize and colonize in the brain liver in lungs [198]. Identification of metastatic drivers in these cells revealed several highly expressed genes in these metastatic cells in which their gene ontologies suggested a process enriched for cell migration and locomotion (metastatic spread) [198]. Among the highly expressed genes, asparagine synthetase was most clinically relevant. In vitro and in vivo experiments revealed that increased levels of bioavailable asparagine supported metastasis in TNBCs [198]. While there was no effect on growth of primary tumors, knocking down asparagine synthetase, treatment with L-asparaginase, or dietary restriction of asparagine significantly reduced metastasis [198]. On the other hand, enhancing asparagine synthetase in TNBCs promoted metastasis [198]. The rate of invasion was proportionally increased with the bioavailability of asparagine, which in turn showed an upregulation of EMT-up genes and asparagine-enriched EMT proteins (Twist1) and was involved in EMT with a decrease in the levels of E-cadherin, thereby promoting EMT and supporting metastasis (Figure 5) [198,344,345]. It is, therefore, recommended that TNBC patients cut down on their dietary intake of asparagine while undergoing chemotherapy [198].

## 6. Observations, Inferences and Concluding Remarks

TNBCs constitute 10–24% of all breast cancers, are most aggressive, and possess higher metastatic potential when compared to other types of breast cancer which are positive for either one or a combination of the ER, PR, or HER2 receptors [8,9,346,347]. Since TNBCs do not respond to hormonal/HER2 targeted therapies due to lack of target receptors, and since no specific promising therapeutic targets were identified, the only treatment modality available for such “no-option” breast cancer patients is the administration of cytotoxic chemotherapeutic drugs [8,9,12,348,349]. Moreover, TNBCs are heterogeneous in nature, show early recurrence, develop resistance, metastasize rapidly, and are associated with a poor prognosis in affected individuals [7,10]. As with many other cancers, using classical chemotherapeutic drugs such as anthracyclines, cyclophosphamides, taxanes, and platinum-based compounds for the treatment of TNBCs is associated with higher incidence of toxic side effects and induced resistance [9]. In this regard, several naturally occurring compounds that are efficient anticancer drugs with much lesser side effects were identified [22]. Additionally, using these phytochemicals in combination with classical anticancer drugs sensitized cancers and reduced the effective dosage of the cytotoxic drug required to bring about anticancer effect and showed improved efficacy with fewer adverse side effects [22].

Here we have linked dysregulation of key signaling pathways (such as the Wnt/β-catenin, the Notch, the NF-κB, the PI3K/Akt/mTOR, and the Hh pathways) and their components to the altered pathological gene and protein expression that contributes to cancer occurrence, progression, growth, invasiveness, metastatic potential, and development of resistance in TNBCs. The dysregulation of these key pathways supported key cellular processes of cell growth, proliferation and migration, inflammation, immunity, angiogenesis, EMT, and metastasis, while suppressing apoptosis in TNBCs. Therefore, targeting the molecular components responsible for aberrant signaling patterns and reversal of the altered processes (by suppression of cell growth, proliferation, migration, inflammation, immunity, angiogenesis, EMT, and metastasis, and activation of apoptosis) in TNBCs should prove to be beneficial in efficiently treating the disease. The natural compounds that we have discussed (luteolin, chalcone, piperine, deguelin, quercetin, etc.) efficiently target these dysregulated pathway components in vitro and in vivo; suppress pro-survival factors, proliferation, migration, and invasion; and enhance pro-apoptotic molecules with few apparent side effects, and can therefore be used safely as anticancer drugs in the treatment for TNBCs.

While some compounds showed specificity (e.g., cyclopamine showed specificity for the Hh signaling pathway), most of the compounds (Table 2) have multiple targets in the different cell signaling pathways in TNBCs. These observations open new areas of research on how to best use these natural compounds in combination with either other phytochemicals or with conventional anticancer drug to improve efficacy of treatment and reduce side effects. Due to the heterogeneous nature of TNBCs and lack of knowledge of specific targets, it would be advantageous to use the multi-targeting nature of phytochemicals and use them in combinations as well. For example, while luteolin targeted Wnt/β-catenin [156,162], Notch signaling [163,164], PI3K/Akt [156], and NF-κB [156] signaling mechanisms, chalcones targeted Wnt/β-catenin [172] and MAPK [205] pathways and deguelin targeted Wnt/β-catenin [218], PI3K/Akt/mTOR [176,220], NF-κB [176], and p38 MAPK [176] signaling mechanisms. Moreover, since there is an obvious overlap in the pathways targeted by the different compounds, using them in combination should be more effective in terms of treatment (to target multiple aberrant pathways) and could help to overcome possible resistance. Additionally, these natural compounds have shown beneficial effects in combination treatments along with classical anticancer drugs [350]. This strategy improves the efficacy of anticancer treatment, reduces side effects, and sensitizes the resistant phenotype to chemotherapy [351]. Combinations of phytochemicals such as resveratrol and quercetin [266], resveratrol and curcumin [236], and curcumin and quercetin [237] were more efficient in the treatment of cancers, including breast cancers, when compared to using these compounds alone for therapeutic purposes. Paclitaxel in combination with luteolin increased cell death in TNBC by suppressing the Notch4 signaling pathway [163]. Efficacy of anticancer drugs cisplatin, 5-fluorouracil, and 4-hydroxycyclophosphamide (metabolite of cyclophosphamide) were enhanced in combinatory treatment with fisetin for TNBC treatment [246]. In MDA-MB-231 cells, rutin enhanced cytotoxicity of cyclophosphamide and methotrexate and restored chemosensitivity in resistant TNBCs by downregulating the activity of P-gp and BCRP pumps [242].

While identifying natural compounds promising for the treatment of TNBCs, other phytochemicals that have the potential to promote and support tumorigenesis and metastasis (e.g., Capsaicin, Asparagine: Table 2) and those compounds which may have a biphasic “hormetic” response (both cancer growth promoting and inhibitory properties) depending on the concentration/dosage used (e.g., resveratrol, genistein: Table 2) were also noted. Asparagine acts as an amino acid exchange factor regulating the uptake of other amino acids such as arginine, histidine, and serine into the cell and increases protein and nucleotide synthesis through the activation of mTORC1, which in turn favors and promotes TNBC cell proliferation [198,341]. Increase in the bioavailability of asparagine lead to an upregulation of EMT-up genes and overexpression of asparagine-enriched EMT proteins (such as Twist1) with a decrease in the levels of E-cadherin, thereby promoting EMT and supporting TNBC metastasis [198]. Capsaicin exhibited EGFR signaling-mediated co-carcinogenic effects in chemically induced skin cancer [311,316], although capsaicin treatment-related adverse effects in breast cancer studies have not been reported. “Hormesis”, defined as “*a dose-response relationship phenomenon characterized by low-dose stimulation and high-dose inhibition independent of the chemical/physical agent or any biological model*” emphasizes the impact of different dosages/concentrations in the assessment and selection of compounds as chemotherapeutic agents [352]. In human tumor cells and endothelial cells, varying doses (0.1–100.0 µg/mL) of resveratrol showed largely different effects [274]. While low doses (0.1–1.0 µg/mL) of resveratrol enhance cell proliferation and promoted cell survival, higher doses (10.0–100.0 µg/mL) induced apoptosis and decreased mitotic activity in cells [274]. Similar observations were made in resveratrol-treated human natural killer cells [273]. Genistein showed a bi-phasic concentration-dependent effect in breast cancer cells [338]. Overall, although many natural compounds are relatively safe, their use as cancer preventive or anticancer agents (with respect to dosage, route of administration, duration of treatment, and number of exposures) must be scientifically addressed and rooted in efficient, well-characterized studies.

Natural compounds can thus effectively be recommended for supporting the therapy of various cancers, including TNBCs. Here, we highlight the pathways and possible molecular targets in TNBCs and the anticancer potential of several natural compounds that can target these dysregulated pathways. It must be noted that while a particular concentration or dosage of the phytochemical may possess anticancer properties, a different concentration/dosage of the same phytochemical may show deleterious effects. Moreover, the effect of the same compound, even at the same concentration/dosage, may vary between different types of cancers. Hence, the importance of relating the effectiveness of a compound in the treatment of TNBCs to a specific dosage/concentration of the compound used is emphasized. There are still abundant natural resources around the globe that remain to be explored for their medicinal/anticancer potential. These untapped resources can be made available to researchers through crowdsourcing projects that involve the participation of non-scientist volunteers, similar to the one that led to the identification of maximiscin as a potential anticancer drug [353]. It is, however, necessary that more anticancer studies be carried out on the active principles or on pure forms of a natural compound to understand its true potential, mainly because the effect and efficacy of compounds in its pure form may be different from its naturally intact form.

## 7. Future Perspective

Carcinogenesis, a complex and multistep process, involves the cumulative effect of intracellular events that affect key cellular processes and pathways such as proliferation, differentiation, invasion, and survival, driven by genetic mutations and epigenetic alterations. Additionally, certain modifiable risk factors such as sedentary life styles, excessive alcohol consumption, and certain dietary habits increase the risk of developing cancers. Therefore, maintaining a healthy life style and inclusion of a healthy diet is key to the prevention of many lifestyle-related diseases, including cancer. We have reviewed and discussed the potential of using plant-derived compounds in the treatment of triple-negative breast cancers. Although certain plant derivatives possess carcinogenic effects, it is evident that many plant-derived substances have the potential to treat different forms of cancer or even prevent the occurrence of cancer.

Epigenetic alterations affect normal gene regulation and adversely affect normal cellular processes related to cell cycle, DNA repair, cell growth, differentiation, and apoptosis. Since epigenetic changes appear early in the development of cancer and are representative of cancer initiation, these epigenetic alterations are promising targets for anticancer interventions. In this regard, plant/naturally derived compounds that alter the epigenetic machinery have shown promise. More studies are required to draw information on dietary and epigenetic implications of food rich in phytochemicals of therapeutic importance in cancer treatment and prevention. Additionally, the efficacy of a compound in its pure form may be different from its naturally intact form. This is one of the reasons for failed translation of in vitro to in vivo studies. In-depth studies on phytochemicals as leading compounds for anticancer treatment require correct translational strategies to draw meaningful outcomes and to avoid any impending drug trial failures.

Different plant-derived compounds target different and diverse cancer-related signaling pathways. A plant derivative that is effective at a particular dosage for the treatment of one type of cancer may not be as efficient in the treatment of other forms of cancer owing to the complexity and diversity among different cancers. It is, therefore, necessary to identify a unified mechanism which can be attributed to and one which can be targeted for the treatment of many different cancers. It is also necessary that the active principles in crude plant extracts be clearly identified and, if possible, modified to increase their potency in the treatment of cancer. Additionally, the anticancer effects of plant derivatives were highly dose/concentration specific. While higher doses of a plant derivative may inhibit cancer signaling pathways, suppress tumor growth, and prove to be cytotoxic, lower concentrations of the same compound may support cell growth and proliferation. Therefore, detailed pharmacology/pharmacokinetics studies are warranted to determine the dosage for a plant-derived compound within the therapeutic window, the route of administration, and the required number of interventions to bring about the best possible anticancer effect with the least adverse side effects.

Although in vitro and in vivo studies have proven the anticancer potential and efficacy of natural compounds, very few of these compounds show comparable or positive results in clinical trials. One key aspect hindering the success of clinical trials is the existence of CSCs and metastasis which are critical problems that adversely affect patient survival. The ability of CSCs (also called tumor-initiating cells) to self-renew themselves and differentiate into heterogeneous multi-lineages of cancer cells may hold the key to tumor progression, drug resistance and cancer relapse. Targeting CSCs through specifically targeting its cell surface markers, stem-cell-specific signaling pathways, pathways shared by CSCs and non-CSCs and CSC metabolism would prove beneficial in combating metastasis, drug resistance and cancer relapse. Among the plant-derived natural anticancer compounds that we have reviewed there are compounds which can target multiple pro-oncogenic and anti-apoptotic pathways. A better understanding on how the CSCs respond to these multi-targeting plant-derived natural anticancer compounds may provide insight into combinatory therapeutic strategies that are more efficient, improve the quality of life and survival of affected individuals. Additionally, the bioavailability of drugs in an in vivo setting is a critical factor in determining the efficacy of a drug. Therefore, novel drug delivery options (nanoparticles/nanomedicine) are needed to improve their bioavailability, to specifically target cancer cells, and to minimize side effects. Additionally, owing to the multiple targets of these natural compounds, studies are warranted to explore the use of phytochemical-phytochemical or phytochemical-anticancer drug combinations to improve efficacy of treatment and reduce side effects.

## Figures and Tables

**Table 1:** Molecular subtyping of triple negative breast cancers (TNBC) based on distinct gene ontology and differential gene expression: TNBC is highly heterogeneous in gene expression. Using cluster analysis, Lehmann classified TNBC into six subtypes. Characteristics of each subtype showing differential gene expression and its representative cell line model for each subtype are provided in the table. Molecular classification helps in the systematic selection of drugs based on biomarkers and enables better prediction of clinical trials.

Subtypes	Characteristics (Based on Gene Ontology and Differential Gene Expression)	Cell Lines
BL1 (Basal like-1)	Enriched cell cycle components DNA replication reactome, G2 cell-cycle pathway, RNA polymerase and G1 to S cell cycle Elevated DNA damage response (ATR/BRCA) High proliferation rate (Ki-67 mRNA) Respond to cisplatin/taxanes	HCC2157 HCC1599 HCC1937 HCC1143 HCC3153 MDA-MB-468 HCC38
BL2 (Basal like-2)	Activated EGF pathway, NGF pathway, MET pathway, Wnt/β-catenin pathway, and IGF-1R Activated glycolysis and gluconeogenesis	SUM149PT CAL851 HCC70 HCC1806 HDQ-P1
IM (Immunomodulatory)	Activated immune cell signaling (TH1/TH2 pathway, NK cell pathway, B cell receptor [BCR] signaling pathway, DC pathway, and T cell receptor signaling), Activated cytokine signaling (IL-12 pathway, and IL-7 pathway) Activated antigen processing and presentation Activated immune signaling pathway (NFKB, TNF, and JAK/STAT signaling)	HCC1187 DU4475
M (Mesenchymal like)	Enhanced expression of genes involved in motility Heightened ECM receptor interaction Activated cell differentiation pathways (Wnt, TGF-β) and genes associated with epithelial-to-mesenchymal transition (EMT).	BT-549 CAL-51 CAL-120
MSL (Mesenchymal Stem Cell-like)	Low levels of proliferation genes and enriched expression of stem cell-associated genes Activated cell motility (Rho pathway), Cellular differentiation, and growth pathways (ALK pathway, TGF-β signaling and Wnt/β-catenin pathway Altered genes linked to EGFR, PDGF, Calcium signaling, G-protein coupled receptor, ERK1/2 signaling, ABC transporters and adipocytokine signaling Enriched in genes involved in angiogenesis	Hs578T MDA-MB-157 MDA-MB-436 MDA-MB-231
LAR (Luminal Androgen Receptor)	Heavily enriched in hormonally regulated pathways including steroid synthesis, porphyrin metabolism, and androgen/estrogen metabolism. AR mRNA is highly expressed.	MDA-MB-453 SUM185PE HCC2185 CAL-148 MFM-223

**Table 2:** Natural substances from plants/fungus indicated in TNBC treatment. Natural compounds mitigate tumor growth by modulating different cell signaling pathways. This table provides an overview of the selected compounds, their potential targets, the signaling pathways involved and experimental models used. Also included is Asparagine, an amino acid and natural compound with pro-carcinogenic effect in TNBCs. Certain non-TNBC cell line (such as MCF-7, T47D, SKBR3 and BT474) related studies have also been mentioned in the table. While the MCF-7 and T47D cells express both the estrogen receptor (ER) and progesterone receptor (PR), the SKBR3 cells express only the human epidermal growth factor receptor 2 (HER2) and are devoid of ER or PR. On the other hand, the BT474 cells express all the three (ER, PR and HER2) receptors. Breast cancers devoid of these three receptors are classified as TNBCs (MDA-MB-231, MDA-Mb-468, MDA-MB-435, BT20, SUM149PT, SUM159PT). LM2-4175 cells indicate MDA-MB-231 TNBC cells derived from lung metastatic sites. 4T1 is a breast cancer cell line derived from the tumors of the mammary glands of mice.

Compound	Chemistry	Source	Conditions Used for	In Vitro	In Vivo	Targets/Markers	Signaling
**Natural Compounds: Having Anti-Cancer Properties in TNBC and Non-TNBC Cell Lines**							
(A) Luteolin	Flavonoid (Figure 2A)	Broccoli, Green chilli, Onion leaves, Carrot, Radish, Celery [168]	Hypertension, inflammatory disorders and cancer [156]	MDA-MB-231, LM2-4175, MDA-MB-435, BT-549	Xenograft	Vimentin, Snail, Slug β-catenin [162] VEGFR2 [161] MMP-2/9 Notch-1, Hes-1, Cyclin D1	PI3K/Akt, MAPK/ERK1/2, STAT3 [169] Notch [164]
(B) Chalcones	Flavonoid (Figure 2B)	Tomatoes, Shallots, Beans, Citrus, Apples	Asthma, gastric ulcer, skin diseases, parasitic infections [170]	MDA-MB-231, BT-549, MDA-MB-468	MDA-MB 231/4mRL.luc2 (SCID) mice	Cell cycle, NF-κB, p65, p38, Hsp90 [171], Wnt/β-catenin, Bcl2 [172]	Wnt/β-catenin, VEGF/VEGFR2
(C) Piperine	Alkaloid (Figure 2C)	Pepper	Pain, chills, fever, reduces blood cholesterol	MDA-MB-231, MDA-MB-468, T47D, MCF-7	MDA-MB-468 (NOD/SCID), 4T1-luc mouse TNBC	TRAIL, MMP 2 and 9 [173], survivin, p65 [174], cell cycle components	ERK1/2, p38 MAPK and Akt [175]
(D) Deguelin	Flavonoid (rotenoid) (Figure 2D)	Natural insecticides	Insecticide piscicide	BT474, T47D, MDA-MB-231, BT-549, BT20, MCF-7	MDA-MB-231 (athymic-mouse)	β-catenin, cyclin D1, XIAP, survivin, EGFR and c-Met	EGFR [176]
(E) Quercetin (F) Rutin	Flavonoid (Figure 2E) (Figure 2F)	Apples, Onions	Cardio-vascular, common cold, allergy	MDA-MB-157, MDA-MB-231, MDA-MB-468,	C3(1)/SV40Tag transgenic mouse	β-catenin, Foxo3A FASN [177] c-Met, p51, p21 and GADD45	Wnt/β-catenin [177] PI3K/ERK/MAPK [178]
(G) Fisetin	Flavonoid (Figure 2G)	Apples, Onions, Kiwi, Cucumber	Ischemic stroke neuroprotective [179] anticancer	MDA-MB-231, MDA-MB-468, MDA-MB-157, SKBR3, MCF-7	xenograft	Bid, Bad, Bak, Bax Aurora B kinase	MAPK/ERK1/2 [180] PI3K/Akt/mTOR NF-κB
(H) Resveratrol	Phytoalexin (Figure 2H)	Red grapes, Blueberries Raspberries	Hyperlipidemia diabetes, atherosclerosis	MDA-MB-435, MDA-MB-231	MDA-MB-231 xenograft	GF-1, MMP2, S6 ribosomal protein, MED28, VEGF	EGFR/PI3K/Akt MAPK [181,182]
(I) Curcumin	Phytopolyl-phenol (Figure 2I)	Turmeric	Food additive, cosmetics, Neuro-degenerative diseases, arthritis	MDA-MB-231, MDA-MB-468, SUM149 PT, SUM159PT	MDA-MB-231 xenograft	VEGFR2/3, EGFR [183,184], Rac1, NF-kB, Akt [185], p53	NF-κB [25] EMT
(J) Maximiscin	Polyketide-shikimate-NRPS-hybrid metabolite (Figure 2J)	Tolypocladium sp in co-culture with bacteria	Data not available	MDA-MB-468	MDA-MB-468 xenograft-	p53, Chk-1 and Chk-2 [186]	DNA damage response
(K) Cyclopamine	Steroidal jerveratrum alkaloid (Figure 2K)	Corn lily	Hypertension, Cardiac diseases, Psoriasis, Basal cell carcinoma, Teratogenic	MDA-MB-231 (is resistant to cyclopamine due low Smo expression) [187], MDA-MB-435	Mouse 4T1	SMO, GLI1 cyclin D1, NF-κB, MMP2 and MMP9 [188]	Hedgehog [189] MAPK/ERK [188]
(L) Capsaicin	Alkaloid (Figure 2L)	Chilli pepper	Pain	MDA-MB-231, BT-474, SKBR3	MDA-MB-231 xenograft	c-Src, FAK and Paxillin MMP2 and MMP9 [190] cyclin D1	EGFR/HER-2 [191]
(M) Genistein	Isoflavanoid (Figure 2M)	Soybeans	Helminthic infection, osteoporosis, cardiovascular diseases, menopause cancer [192]	MDA-MB-231, MDA-MB-468, T47D, MCF-7	MDA-MB-231 xenograft	MMP-9 p21 VEGF TGF-β [193] Bcl-2, Bax, p53 [194]	Hedgehog [195] PTEN/PI3K/Akt (inhibition of mammosphere formation) [196] EGFR [197]
**Natural Compound: Having Pro-Carcinogenic Effect in TNBC**							
(N) Asparagine	Non-essential amino acid (Figure 2N)	Asparagus Potatoes, Legumes, Beef, Egg, Fish	Biosynthetic role	4T1, MDA-MB-231	MDA-MB-231 Mouse 4T1 xenograft	Twist E-cadherin [198]	EMT [198]

## Abbreviations

AIF	Apoptosis-Inducing Factor
Akt	Serine/threonine-specific protein kinase or Protein Kinase B
AMPK	5′ AMP-activated Protein Kinase
Ang1	Angiopoietin-1
APC	Adenomatous Polyposis Coli
AR	Androgen Receptor
BCRP	Breast Cancer Resistant Protein
BRCA1	Breast Cancer 1, early onset
CD	Cardamonin
Chk 1/2	Check point kinases 1/2
CK1	Casein Kinase-1
COX2	Cyclooxygenase-2
CSCs	Cancer Stem Cells
CXCR4	C-X-C chemokine Receptor type 4
DHh	Desert Hedgehog
DLL	Delta-like Ligand
ECD	Extra Cellular Domain
EGFR	Extracellular Growth Factor Receptor
EMT	Epithelial-Mesenchymal Transition
ER	Estrogen Receptor
ER-α	Estrogen Receptor-alpha
ERK1/2	Extracellular signal-Regulated Kinase-1/2
FABP5	Fatty Acid-Binding Protein 5
FAK	Focal Adhesion Kinase
Fas	First apoptosis signal
FasL	Fas Ligand
FasR	Fas Receptor
FGF	Fibroblast Growth Factor
FZD_7_	Frizzled Receptor 7
GADD45	Growth Arrest and DNA Damage-inducible 45
Gli	Glioma-associated oncogene
GRB2	Growth factor Receptor Binding protein 2
GSK-3β	Glycogen Synthase Kinase-3β
GTPs	Green Tea Phenols
HER2	Human Epidermal growth factor Receptor 2
HGF	Hepatocyte Growth Factor
Hh	Hedgehog
HIF1	Hypoxia Inducible Factor 1
IHh	Indian Hedgehog
IL1	Interleukin-1
JAG1/2	Jagged 1/2
LAR	Luminal Androgen Receptor
LRP5/6	Low-density lipoprotein Receptor-related Protein-5/6
MAM	Mastermind
MAPK	Mitogen-Activated Protein Kinase
MAPKAP	Mitogen-Activated Protein Kinase Activated Protein
MAPKAP Kinase	Mitogen-Activated Protein Kinase Activated Protein Kinase
MCP	Monocyte Chemoattractant Protein
MDR1	Multidrug Resistance Protein-1
MEK1/2	Mitogen-activated protein kinase ERK kinase 1/2
MMP 2/7/9	Matrix metalloproteinase 2/7/9
NEXT	Notch Extracellular Truncation
NF-κB	Nuclear factor kappa-light-chain-enhancer of activated B cells
NICD	Notch Intracellular Domain
P-gp	P-glycoprotein
PARP	Poly (ADP-ribose) Polymerase
PDGF	Platelet-Derived Growth Factor
PIP2	Phosphatidyl-Inositol biphosphate
PR	Progesterone Receptor
Ptch	Patched
PTEN	Phosphatase and tensin homolog
RHD	Rel Homology Domain
RSK	Ribosomal s6 kinase
SHh	Sonic Hedgehog
Smo	Smoothened
TCF/LEF	T-cell factor/Lymphoid Enhancing Factor
TGFβ	Transforming Growth Factor-b
TLR4	Toll-like Receptor 4
TNBCs	Triple-Negative Breast Cancers
TNF-α	Tumor Necrosis Factor-α
TNFR	Tumor Necrosis Factor Receptor
TRAIL	Tumor Necrosis Factor-Related Apoptosis-Inducing Ligand
TSC 1/2	Tuberous Sclerosis Complex-1/2
Twist 1	Twist-related protein-1
VEGF	Vascular Endothelial Growth Factor
VEGFR2	Vascular Endothelial Growth Factor Receptor-2
VRK1	Vaccinia-Related Kinase-1
WISP3	Wnt1-Inducible Signaling Protein-3
XIAP	X-linked Inhibitor of Apoptosis Protein
